# MTSS1 is downregulated in nasopharyngeal carcinoma (NPC) which disrupts adherens junctions leading to enhanced cell migration and invasion

**DOI:** 10.3389/fcell.2023.1275668

**Published:** 2023-10-18

**Authors:** Shixing Zheng, Xiaoxia Wang, Liudmila Matskova, Xiaoying Zhou, Zhe Zhang, Elena Kashuba, Ingemar Ernberg, Pontus Aspenström

**Affiliations:** ^1^ Department of Microbiology, Tumor and Cell Biology, Karolinska Institutet, Stockholm, Sweden; ^2^ ENT Institute and Department of Otorhinolaryngology, Eye & ENT Hospital, Fudan University, Shanghai, China; ^3^ Scientific Research Centre, Life Science Institute, Guangxi Medical University, Nanning, China; ^4^ Department of Otolaryngology-Head and Neck Surgery, First Affiliated Hospital of Guangxi Medical University, Nanning, China; ^5^ RE Kavetsky Institute of Experimental Pathology, Oncology and Radiobiology of National Academy of Sciences of Ukraine, Kyiv, Ukraine; ^6^ Rudbeck Laboratory, Department of Immunology, Genetics and Pathology, Uppsala University, Uppsala, Sweden

**Keywords:** invasion, metastasis, MTSS1, nasopharyngeal carcinoma, adherens junctions, I-BAR domain

## Abstract

Loss of cell–cell adhesions is the indispensable first step for cancer cells to depart from the primary tumor mass to metastasize. Metastasis suppressor 1 (MTSS1) is frequently lost in metastatic tissues, correlating to advanced tumor stages and poor prognosis across a variety of cancers. Here we explore the anti-metastatic mechanisms of MTSS1, which have not been well understood. We found that MTSS1 is downregulated in NPC tissues. Lower levels of MTSS1 expression correlate to worse prognosis. We show that MTSS1 suppresses NPC cell migration and invasion *in vitro* through cytoskeletal remodeling at cell–cell borders and assembly of E-cadherin/β-catenin/F-actin in adherens junctions. The I-BAR domain of MTSS1 was both necessary and sufficient to restore this formation of E-cadherin/β-catenin/F-actin–mediated cell adherens junctions.

## 1 Introduction

Undifferentiated nasopharyngeal carcinoma (NPC) is an epithelial cancer that arises from cells lining the surface of the nasopharynx. The pathogenesis depends on a challenging complex of risk factors, including polygenic susceptibility, Epstein–Barr virus (EBV) infection, and other environmental factors (Chen et al., 2019). Nasopharyngeal carcinoma has the highest metastasis rate upon first diagnosis among all head and neck cancers, ranging from 9.0% to 15.5% (Yeh et al., 2006; Liu et al., 2019; Qu et al., 2020). Up to 15% of patients with NPC treated with radical radiotherapy with or without chemotherapy still develop local recurrence after the initial therapy (Yeh et al., 2006; Lee et al., 2015). Early detection of NPC would offer increased chances for successful treatment with curative intent (Schiffman et al., 2015; Crosby et al., 2020).

Metastasis starts with the release of cells from the primary tumor tissue. The cells then disseminate through tissue via lymph and blood vessels, are carried through the general blood circulation into a distant tissue to form secondary tumors (Chaffer and Weinberg, 2011; Suhail et al., 2019). To maintain the integrity of the epithelial sheet, epithelial cells are linked to their neighboring cells through cell–cell junctions, resulting in control of cell migration and cell communication (D'Souza-Schorey, 2005). Cell–cell junctions include tight junctions, adherens junctions, desmosomes and gap junctions. Among these, the adherens junctions form the strong lateral cell–cell connections, which are especially important for the self-organization of normal epithelia. Adherens junctions are often weakened in epithelial tumors (D'Souza-Schorey, 2005). Mature epithelial adherens junctions are formed by the adhesion complex at the plasma membrane, consisting of epithelial cadherin (E-cadherin), along with the catenins that bind the complex to the filamentous actin (F-actin) in the cytosol (Harris and Tepass, 2010). In many epithelial cancers, the loss of E-cadherin results in defects in adherens junctions and activation of epithelial–mesenchymal transition (EMT) and metastasis (Knights et al., 2012; Bruner and Derksen, 2018). *β*-Catenin and p120 catenin are the most important catenins in the formation of adherens junctions (Hartsock and Nelson, 2008; Niessen and Gottardi, 2008). They fulfill different functions depending on whether they are localized at cell-cell junctions or in the cell nucleus. Catenins localized to cell-cell junctions contribute to the formation and stabilization of adherens junctions, and can thereby serve as metastasis suppressors (Gavert and Ben‐Ze’ev, 2007; Reynolds and Roczniak-Ferguson, 2004).

Metastasis suppressor 1 (*MTSS1*) was originally named “missing in metastasis” (*MIM*) because it was not expressed in invasive, metastatic bladder cancer cell lines (Lee et al., 2002). Wild-type MTSS1 contains 759 amino acid residues, with a C-terminal “Wiskott–Aldrich syndrome protein homology 2” (WH2) domain and an N-terminal “inverse Bin–Amphiphysin–Rvs” (I-BAR) domain (Machesky and Johnston, 2007). The C-terminal WH2 domain interacts with actin, whereas the I-BAR domain confers binding to phosphoinositide-rich lipid bilayers. The I-BAR domain introduces a curvature in lipid bilayers, such as at the plasma membrane. In this way, I-BAR domains can induce the formation of protrusions at the cell surface (Frost et al., 2009; Zhao et al., 2011; Carman and Dominguez, 2018). At cell–cell junctions, it is likely that the WH2 and I-BAR domains act in concert to sense the curvature of the loosely associated plasma membranes of two adjacent cells, and to organize F-actin to keep the junctional structure intact (Maddugoda et al., 2011).

In the present study, we identified low expression level or lack of MTSS1 as a component of NPC cell dissemination. Using a panel of antibodies against the components of cell–cell junctions followed by immunofluorescence microscopy, we show that MTSS1 has a key role in the regulation of the integrity of adherens junctions. By gain of function experiments, we show that re-expression of MTSS1 induced E-cadherin and *β*-catenin accumulation, as well as cytoskeletal reorganization, at cell–cell borders. Moreover, re-expression of MTSS1 reduced NPC cell migration, invasion, and motility *in vitro*. Knockdown of MTSS1 in cell lines with high expression logically showed the reversed phenotypic shift. Importantly, our studies show that low MTSS1 expression correlated with worse clinical outcomes in NPC patients. These observations have implications for all cancer types with decreased expression of MTSS1.

## 2 Materials and Methods

Further details of the Materials and Methods are provided in Supplementary Material.

### 2.1 Cell lines and plasmids

Seven EBV-negative NPC-derived cell lines: 5-8F (RRID: CVCL_C528), 6-10B (RRID: CVCL_C529), TW03 (RRID: CVCL_6010), CNE1 (RRID: CVCL_6888), HONE1 (RRID: CVCL_8706), HK1 (RRID: CVCL_7084), and CNE2 (RRID: CVCL_6889) cells, and one EBV-positive NPC cell line, C666-1 (RRID: CVCL_7949), were available to us and included in this study. Vectors encoding Myc-tagged human MTSS1 FL (pRK5Myc-MTSS1; amino acids 1-759), MTSS1 K4D (pRK5Myc-MTSS1 K4D; mutant K149,150,152,153D), MTSS1 △I-BAR (pRK5Myc-MTSS1; amino acids 235-759), and MTSS1 I-BAR (pRK5Myc-MTSS1; amino acids 1-254) were kindly provided by Laura M. Machesky (Beatson Institute, Glasgow, UK). The pCMV-tdTomato plasmid with a similar plasmid backbone, pRK5, was purchased from Addgene (54,642; Watertown, MA, United States) and the pCMV-Myc vector was purchased from Clontech (635,689, Mountain View, CA, United States).

### 2.2 Human specimens

A tissue microarray containing 131 cases of primary NPC individually linked to clinically relevant information was purchased from Outdo Biotech (HNasN132Su01; Shanghai, China). Normal nasopharyngeal epithelium and primary NPC biopsies were obtained from the Department of Otolaryngology-Head and Neck Surgery, First Affiliated Hospital of Guangxi Medical University (Nanning, Guangxi, China). The specimen collection was approved by the Research Ethics Committee of First Affiliated Hospital of Guangxi Medical University (Ref. N° 2016-175, Ref. N° 2021-163) and Regionala Etikprövningsnämnden, Stockholm (Ref. N° 00-312). The specimen collection was undertaken with the understanding and written consent of each subject, and the specimens were anonymized. The study methodologies conformed to the standards set by the Declaration of Helsinki.

## 3 Results

### 3.1 MTSS1 is downregulated in NPC and loss of MTSS1 predicts a poor prognosis for patients with NPC

**FIGURE 1:**
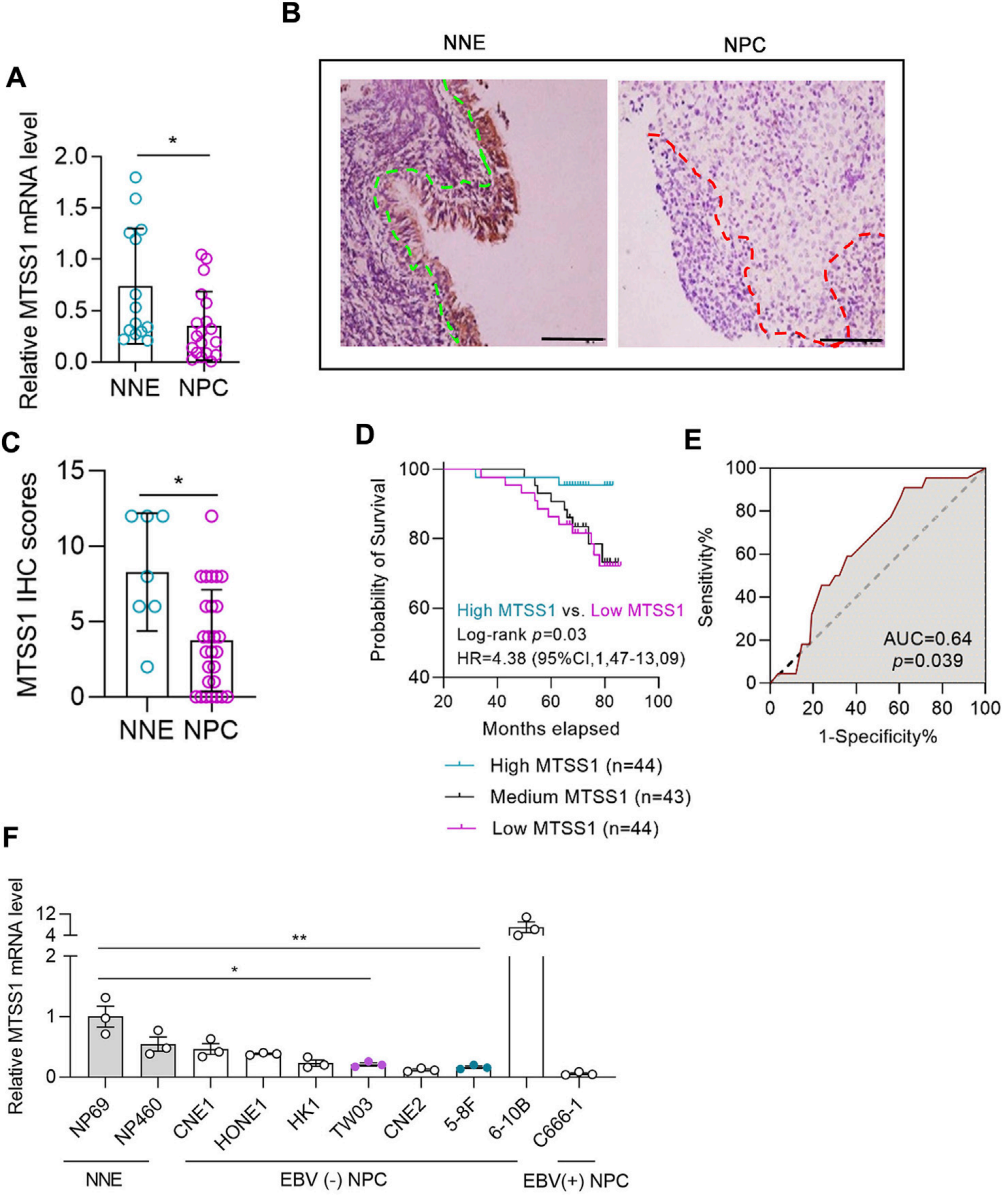
MTSS1 is downregulated in nasopharyngeal carcinoma (NPC). MTSS1 downregulation correlates with the clinicopathological characteristics of patients with NPC. **(A)** RT–qPCR analysis of relative mRNA levels of MTSS1 in normal nasopharyngeal epithelium (NNE) and NPC tissues. **(B)** Representative immunohistochemistry staining of MTSS1 in NNE and NPC biopsies. Green line represents the margin of normal epithelial, while the red line represents the margin of the NPC tissue. Scale bar, 100 μm. **(C)** Immunohistochemistry (IHC) staining scores for MTSS1 expression. **(D)** Kaplan-Meier analysis followed by log-rank test for overall survival of patients with NPC and low (*n* = 44) versus high (n = 44) MTSS1 expression. **(E)** Receiver operating characteristic (ROC) curve of MTSS1 immunohistochemistry scores for surviving versus deceased patients with NPC. **(F)** RT-qPCR analysis of relative MTSS1 expression in two immortalized nasopharyngeal epithelial cell lines (NP460 and NP69) and eight NPC cell lines. Numerical data are given as means ± SEM. *, *p* ≤ 0.05, **, *p* ≤ 0.01 (Unpaired, two-tailed Mann Whitney U test).

Meta-analysis of seven different microarray datasets revealed that MTSS1 was significantly downregulated in NPC tissue compared to normal nasopharyngeal epithelium (NNE) at the transcriptional level (pooled standard mean difference, −0.59; *p* = 0.0009) (Supplementary Figures S1A, B). Downregulation of MTSS1 in NPC was also validated by qPCR (Figure 1A). The mean of MTSS1 mRNA expression level was 0.35 (SEM ± 0.33) in 19 NPC cases and 0.74 (SEM ± 0.56) in the NNE control group (*n* = 14), respectively.

The IHC staining showed that the MTSS1 expression was low in the NPC biopsy tissues compared to controls (Figure 1B), which was underpinned by the semi-quantitative scoring (Figure 1C). In this IHC experiment with 131 NPC cases and 7 NNE controls, the mean expression of MTSS1 was 3.74 (SEM 3.69) in the NPC group and 8.29 (SEM 3.90) in the NNE control group. To investigate the clinical relevance of MTSS1 in patients with NPC, we correlated the MTSS1 expression pattern in NPC with clinically relevant endpoints. The clinical characteristics of the patients with NPC are summarized in Supplementary Table S2 and the workflow is shown in Supplementary Figure S1C.

A higher number of patients at stage IV (10 out of 21, or 48%) showed low MTSS1 expression compared to stage I (4 out of 15, 27%); while conversely less patients at stage IV were characterized by high MTSS1 expression (3 out of 21, 14%) compared to stage I (5 out of 15, 33%). The Kaplan-Meier analysis showed that the patients with low expression of MTSS1 had worse overall survival compared to those with higher levels of MTSS1 (Figure 1D). In this cohort study, the number of the deceased/total NPC patients in the “high” MTSS1 group was 2 out of 44 (<5%), while in “low” MTSS1 group this number increased dramatically to 11 out of 44 (25%). Relating the MTSS1 expression to survival prediction for these 131 patients resulted in an area under the receiver operating characteristic (ROC) curve of 0.64 (Figure 1E). Thus comparing two NPC patients where one has high MTSS1 expression and the other one has low MTSS1 expression, the model predicts a better prognosis (i.e., survival) for patients with high MTSS1 expression in 64% of cases, while lower expression of MTSS1 correlated with a worse prognosis for these patients with NPC.

### 3.2 MTSS1 suppresses nasopharyngeal carcinoma cell migration, invasion, and motility

**FIGURE 2:**
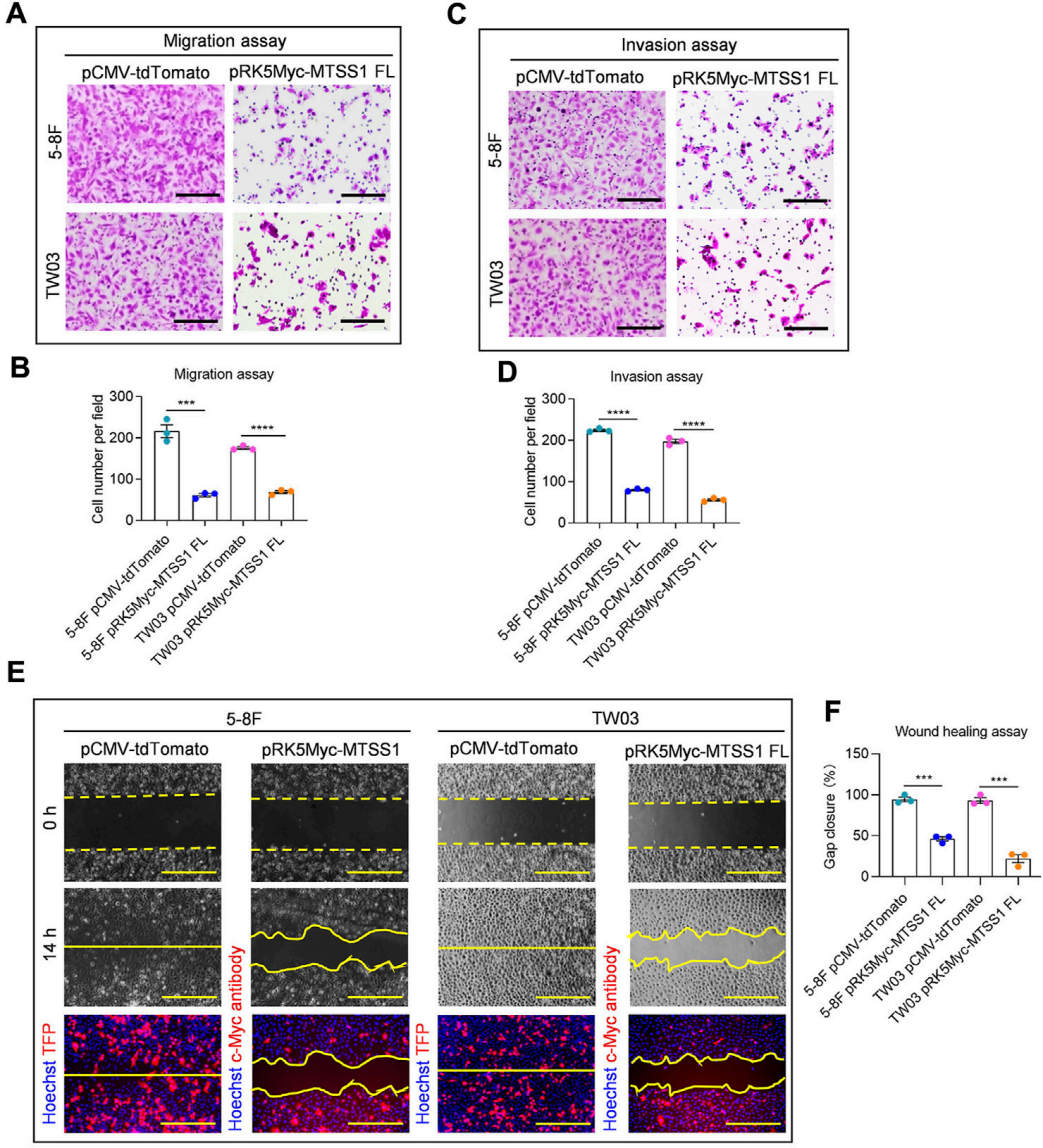
Over-expression of MTSS1 suppresses cell migration, invasion and motility of NPC cells. **(A)** Representative images of cell migration assay. Scale bar, 250 µm. **(B)** Quantification of cell migration. **(C)** Representative images of cell invasion assay. Scale bar, 250 µm. **(D)** Quantification of cell invasion. **(E)** Representative images of cell wound healing assay. Red, TFP (tdTomato fluorescent protein)/MTSS1; Blue, Hoechst. Scale bar, 500 µm. **(F)** Quantification of gap closure measured as percentage of the original wound area. Numerical data are given as means ± SEM. ***, *p* ≤ 0.001, ****, *p* ≤ 0.0001 (*n* = 3 biological replicates/group; Unpaired, two-tailed Student’s *t*‐tests).

Next, we wanted to assess the MTSS1 mRNA expression in NPC-derived cell lines, compared to control cell lines. The MTSS1 expression was quite low in most of the cell lines, the lowest levels of the MTSS1 mRNA were detected in the two NPC cell lines that have been shown to be highly metastatic *in vivo*, the 5-8F and TW03 (Figure 1F). The 5-8F and TW03 cell lines were therefore selected for our *in-vitro* studies. qPCR was performed at 36 h post-transfection of MTSS1, showing that MTSS1 was highly expressed in these 2 cell lines after transfection (Supplementary Figure S1D). The migration and invasion abilities of cells ectopically expressing MTSS1 were evaluated using Transwell migration and Matrigel invasion assays, respectively. There was a significantly decreased migration of the 5-8F and TW03 cells transfected with MTSS1 compared to the control cells (transfected with pCMV-tdTomato) (*p* < 0.005; Figures 2A, B). A similar result was observed in the Matrigel invasion assay; there was a significantly lower invasion by the cells with increased expression of MTSS1 (between 70% and 80%, *p* < 0.005) (Figures 2C, D). To measure collective cell migration, wound healing assays were performed. Again, the cells ectopically expressing MTSS1 had a significantly reduced wound closure capacity compared to the control cells (Figures 2E, F).

### 3.3 MTSS1 induces genes that may contribute to adherens junction assembly

To gain more insight into the inhibitory role of MTSS1 on cell migration, RNA-sequencing was used to identify genes that were affected by increased MTSS1 expression caused by transfection of MTSS1. Pathway enrichment analysis of a rank-ordered gene list was implemented in the GSEA software, to uncover genes that could provide clues to the function of MTSS1 in its role to suppress 5-8F and TW03 NPC cell migration.

Using GSEA on the Reactome pathway gene sets revealed that ectopic MTSS1 expression in the 5-8F and TW03 NPC cells positively correlated with pathways involved in cell junction organization (Supplementary Figure S2A) and adherens junction interactions (Supplementary Figure S2B). Using Gene Ontology molecular function (GOMF) analysis with the GSEA software, we showed that the restored MTSS1 expression affects proteins coupled to the binding of actin monomers (Supplementary Figure S2C) and cadherin (Supplementary Figure S2D) in cell–cell adhesion. Gene Ontology cellular component (GOCC) analysis suggested that MTSS1 affects actin and cadherin binding in the adherens junction (Supplementary Figure S2E) and to the catenin complex (Supplementary Figure S2F). To determine which genes contributed most to these MTSS1 enrichment data, a subset of genes was subjected to so-called leading-edge analysis. The leading-edge analysis showed that the adherens junction component E-cadherin (CDH1) appeared in four subsets (Supplementary Figure S2G), suggesting that expression of E-cadherin correlates with MTSS1 expression. This observation made it relevant to explore further the contribution of E-cadherin to the MTSS1-dependent maintenance of cell adherens junctions.

### 3.4 The I-BAR domain of MTSS1 is sufficient to promote formation of E-cadherin/β-catenin mediated cell adherens junctions

We next analyzed the effects of increased MTSS1 expression on a panel of cell junction components in 5-8F and TW03 cells. It is known that Desmoplakin (DSP) and Integrin beta-4 (ITGβ4) are components of focal junctions and desmosomes, respectively. MTSS1 did not significantly alter the mRNA level of DSP or ITGβ4. Additional cell junctional components were analyzed, however, we did not observe that the expression levels of the tight junction components Claudin-1 (CLDN1), junctional adhesion molecule A (JAM1) or ZO-1 (TJP1) were significantly changed upon MTSS1 expression (Supplementary Figure S3A). In contrast, E-cadherin was significantly upregulated in response to MTSS1 expression in both 5-8F and TW03 NPC cells in line with the qPCR data (Supplementary Figure S3A), while other adherens junction components, such as Catenin alpha-1 (CTNNA1) and Occludin (OCLN) were downregulated by MTSS1 in 5-8F but not in TW03 (Supplementary Figure S3A).

**FIGURE 3:**
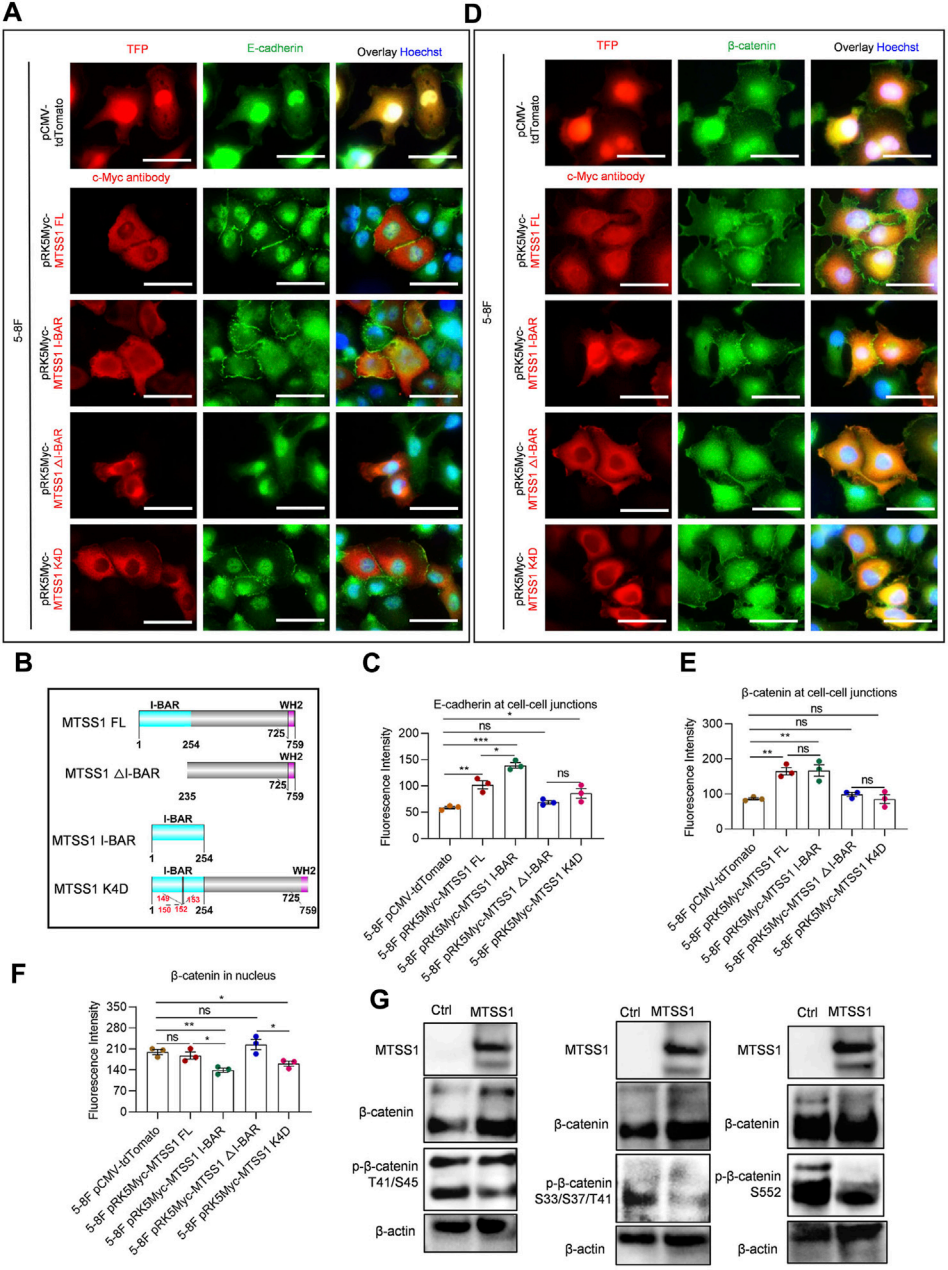
The I-BAR domain is sufficient for MTSS1 to promote formation of E-cadherin/β-catenin–mediated cell adherens junctions. **(A)** Representative immunofluorescence staining of adhering 5-8F NPC cells. Red, TFP (tdTomato fluorescent protein)/MTSS1; green, E-cadherin; Blue, Hoechst. Scale bar, 50 µm. **(B)** Schematic representation of the sequence domains of MTSS1 FL, MTSS1 K4D, MTSS1 △I-BAR, and MTSS1 I-BAR. I-BAR = inverse BAR domain. **(C)** Quantification of immunofluorescence intensity of E-cadherin at adherens junctions, using the ZEN software. **(D)** Representative immunofluorescence staining of adhering 5-8F NPC cells Red, TFP (tdTomato fluorescent protein)/MTSS1; green, *β*-catenin. Blue, Hoechst. Scale bar, 50 µm. **(E, F)** Quantification of immunofluorescence intensity of *β*-catenin at adherens junctions **(E)** and nucleus **(F)**, using the ZEN software. **(G)** Western blot analysis of *β*-catenin phosphorylation levels in response to MTSS1 expression in TW03 cells. Numerical data are given as means ± SEM. *, *p* ≤ 0.05; **, *p* ≤ 0.01; ***, *p* ≤ 0.001; ns, *p* > 0.05 (n = 3 biological replicates/group; Unpaired, two-tailed Student’s *t*‐tests).

We next used immunofluorescence staining to investigate the effects of increased MTSS1 expression on adherens junctions and F-actin distribution in 5-8F. Several cell junction components were analyzed for their subcellular localization, including E-cadherin, *β*-catenin, Claudin-1, JAM1, DSP, and ITGβ4 (Supplementary Figure S3B). Of these proteins, only E-cadherin and *β*-catenin were significantly relocalized in response to MTSS1 expression. Notably, E-cadherin was redistributed to the adherens junctions in response to MTSS1 expression (Figure 3A). We thereafter analyzed which part of MTSS1 was responsible for the redistribution (Figure 3B). Cells expressing the I-BAR domain alone resulted in an even stronger accumulation of E-cadherin at cell adherens junctions (Figures 3A, C; Supplementary Figures S4A, B). Conversely, inactivation of the I-BAR domain by point mutations (MTSS1 K4D) or deletion of the entire I-BAR domain (MTSS1△I-BAR) strongly impaired the re-distribution of E-cadherin (Figures 3A, C; Supplementary Figures S4A, B).

It is well known that *β*-catenin is a dual function protein involved in the formation and organization of cell adherens junctions, while, when translocated to the cell nucleus, it may promote cancer development by regulating gene expression (Gavert and Ben‐Ze’ev, 2007). Expression of full-length MTSS1 induced a significant redistribution of *β*-catenin to adherens junctions in 5-8F and TW03 cells (Figures 3D, E; Supplementary Figures S4C, D). Expression of the I-BAR domain alone cells was sufficient to induce the recruitment of *β*-catenin to the cell adherens junctions (Figures 3D, E; Supplementary Figures S4C, D). When the I-BAR domain (MTSS1△I-BAR and MTSS1 K4D) was inactivated, much less of *β*-catenin localized to the adherens junctions (Figures 3D, E; Supplementary Figures S4C, D). No nuclear *β*-catenin accumulation was seen in cells expressing full-length MTSS1 (Figures 3D, F; Supplementary Figures S4C, D). Although there was no increase in nuclear *β*-catenin or any activation of the Wnt/β-catenin signaling pathway (Supplementary Figure S5A), four phosphatases reported to dephosphorylate beta-catenin and prevent it from nuclear shuttling were both upregulated by MTSS1 in 5-8F and TWO3 cells: protein tyrosine phosphatase receptor type U (PTPRU), protein tyrosine phosphatase receptor type F (PTPRF), protein tyrosine phosphatase receptor type G (PTPRG) and protein tyrosine phosphatase receptor type K (PTPRK) (Supplementary Figure S5C). Interestingly, we could detect a decreased phosphorylation on several sites in response to MTSS1 expression (Figure 3G).

### 3.5 MTSS1 regulates filopodia formation, reduction of lamellipodia, and loss of front-rear polarity

**FIGURE 4:**
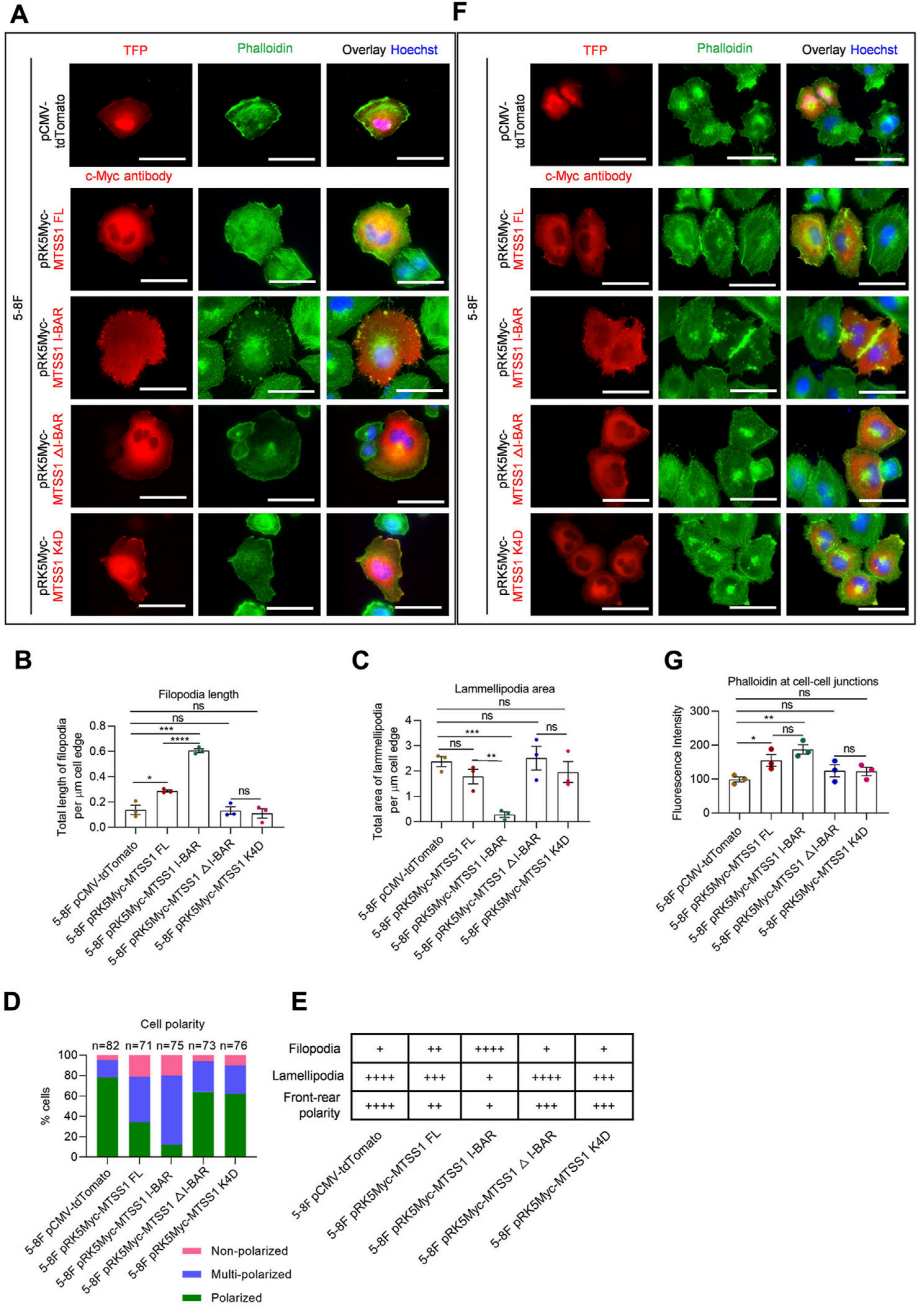
MTSS1 regulates actin dynamics for the formation of cell–cell junctions. **(A)** Representative immunofluorescence staining of adhering 5-8F NPC cells to show the cell morphology. Red, TFP (tdTomato fluorescent protein)/MTSS1; green, phalloidin; Blue, Hoechst. Scale bar, 50 µm. **(B–D)** Quantification of filopodia length **(B)** and lamellipodia area **(C)** along the cell edge, and cell polarity **(D)**. **(E)** Summary of the 5-8F NPC cell morphology. **(F)** Representative immunofluorescence staining of adhering 5-8F NPC cells to show the adherens junctions. Red, TFP (tdTomato fluorescent protein)/MTSS1; green, phalloidin; Blue, Hoechst. Scale bar, 50 µm. **(G)** Quantification of immunofluorescence intensity of F-actin at cell–cell junctions, using the ZEN software. Numerical data are given as means ± SEM. *, *p* ≤ 0.05; **, *p* ≤ 0.01; ***, *p* ≤ 0.001; ****, *p* ≤ 0.0001; ns, *p* > 0.05 (*n* = 3 biological replicates/group; Unpaired, two-tailed Student’s *t*‐tests).

We next analyzed the effects on F-actin organization by increased expression of MTSS1 in 5-8F and TW03 cells. The distribution of F-actin was analyzed using fluorescently labeled phalloidin. Compared to the control cells expressing the control plasmid, the cells expressing full-length MTSS1 showed clear morphological differences, which were characterized by longer filopodia, fewer lamellipodia, and loss of front-rear polarity (Figures 4A–D; Supplementary Figures 6A–D). These changes were similar, but more dramatic, in the cells with increased expression of the I-BAR domain alone (Figures 4A–D; Supplementary Figures 6A–D). In contrast, no significant differences were seen in the cells expressing MTSS1 △I-BAR or K4D (Figures 4A–D; Supplementary Figures 6A–D). The mutant I-BAR domain mimicked the complete deletion of the I-BAR domain in terms of its lack of effects with such morphological consequences. The morphological features of these cell lines with forced expression of the different forms of MTSS1 are summarized in Figure 4E (5-8F cells) and Supplementary Figure S5E (TW03 cells).

We next investigated whether the accumulation of the junction components was concomitant with redistribution of F-actin. Increased expression of full-length MTSS1 resulted in the formation of a broad belt of cortical actin filaments at cell–cell junctions (Figure 4F; Supplementary Figure S5F). Cell–cell junctions between cells expressing MTSS1 I-BAR alone were more prominent than in the cells expressing MTSS1△I-BAR and MTSS1 K4D (Figures 4F, G; Supplementary Figures 6F, G). Thus, the loss of the functional I-BAR domain disrupted the actin filament assembly at cell–cell junctions induced by MTSS1 (Figures 4F, G; Supplementary Figures 6F, G).

### 3.6 MTSS1 suppression of nasopharyngeal carcinoma cell migration, invasion, and motility is dependent on its active I-BAR domain

We next examined whether the I-BAR domain is responsible for the decreased migration, invasion, and motility in MTSS1-expressing 5-8F and TW03 cells. To this end, full-length MTSS1, MTSS1 I-BAR, MTSS1 △I-BAR, and MTSS1 K4D were transfected into 5-8F and TW03 cells. Thereafter, the migratory and invasive capacities were analyzed as before. All of the transfected mutants of MTSS1 inhibited cell migration analyzed by the Transwell migration assay and invasion measured by the Matrigel invasion assay, respectively, if compared to the mock transfected (Supplementary Figures 7, 8A–D). Full-Length MTSS1 and the I-BAR domain had a more pronounced effect on cell migration and invasion compared to those with lack of I-BAR-function MTSS1 △I-BAR and K4D. The I-BAR domain alone had the greatest negative effect on cell migration and invasion in these assays. Compared to MTSS1-negative cells, full length MTSS1 and the MTSS1 I-BAR domain alone clearly suppressed cell migration in the wound healing assay (Supplementary Figures 7, 8E, F), whereas the expression of MTSS1 △I-BAR and MTSS1 K4D did not suppress wound closure (Supplementary Figures 7, 8E, F).

### 3.7 MTSS1 silencing triggers nasopharyngeal cell migration, invasion and motility and impairs adherens junction formation

**FIGURE 5:**
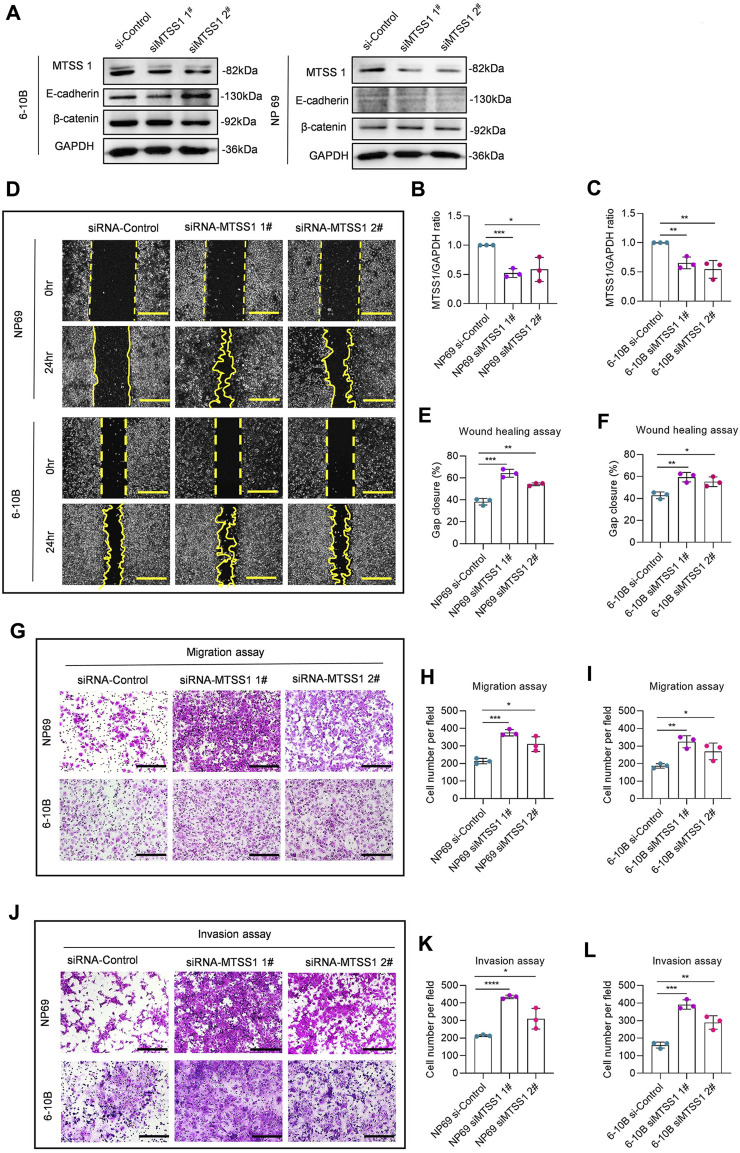
Silencing of MTSS1 suppresses cell migration, invasion and motility of NPC cells. **(A)** Western blot analysis of MTSS1 expression in two epithelial cell lines (NP69 and 6-10B). **(B, C)** Quantification of the efficiency of silencing with two different MTSS1-specific siRNAs by Western Blot. **(D)** Representative images of cell wound healing assay. **(E, F)** Quantification of gap closure. **(G)** Representative images of cell migration assay. **(H, I)** Quantification of cell migration. **(J)** Representative images of cell invasion assay. Scale bar, 250 µm. **(K, L)** Quantification of cell invasion. Numerical data are presented as means ± SEM. *, *p* ≤ 0.05; **, *p* ≤ 0.01; ***, *p* ≤ 0.001; ****, *p* ≤ 0.0001; ns, *p* > 0.05 (*n* = 3 biological replicates/group; Unpaired, two-tailed Student’s *t*‐test).

We next aimed to study if silencing of MTSS1 in a nasopharyngeal epithelial cell (NP69) or low metastatic capacity NPC cell (6-10B) was associated with an increased migratory phenotype. We silenced MTSS1 with two different siRNAs in NP69 and 6-10B cells, which both show a high expression of MTSS1 (Figure 2A), and confirmed a significant decrease in MTSS1 protein as determined by Western blotting and qPCR (Figures 5A–C). We found a significantly increased wound closure activity in cells treated with MTSS1 siRNAs (Figures 5D–F). MTSS1 silencing was also associated with increased migration and invasion in Transwell migration (Figures 5G–I) and Matrigel invasion assays (Figures 5J–L) compared to control conditions.

**FIGURE 6:**
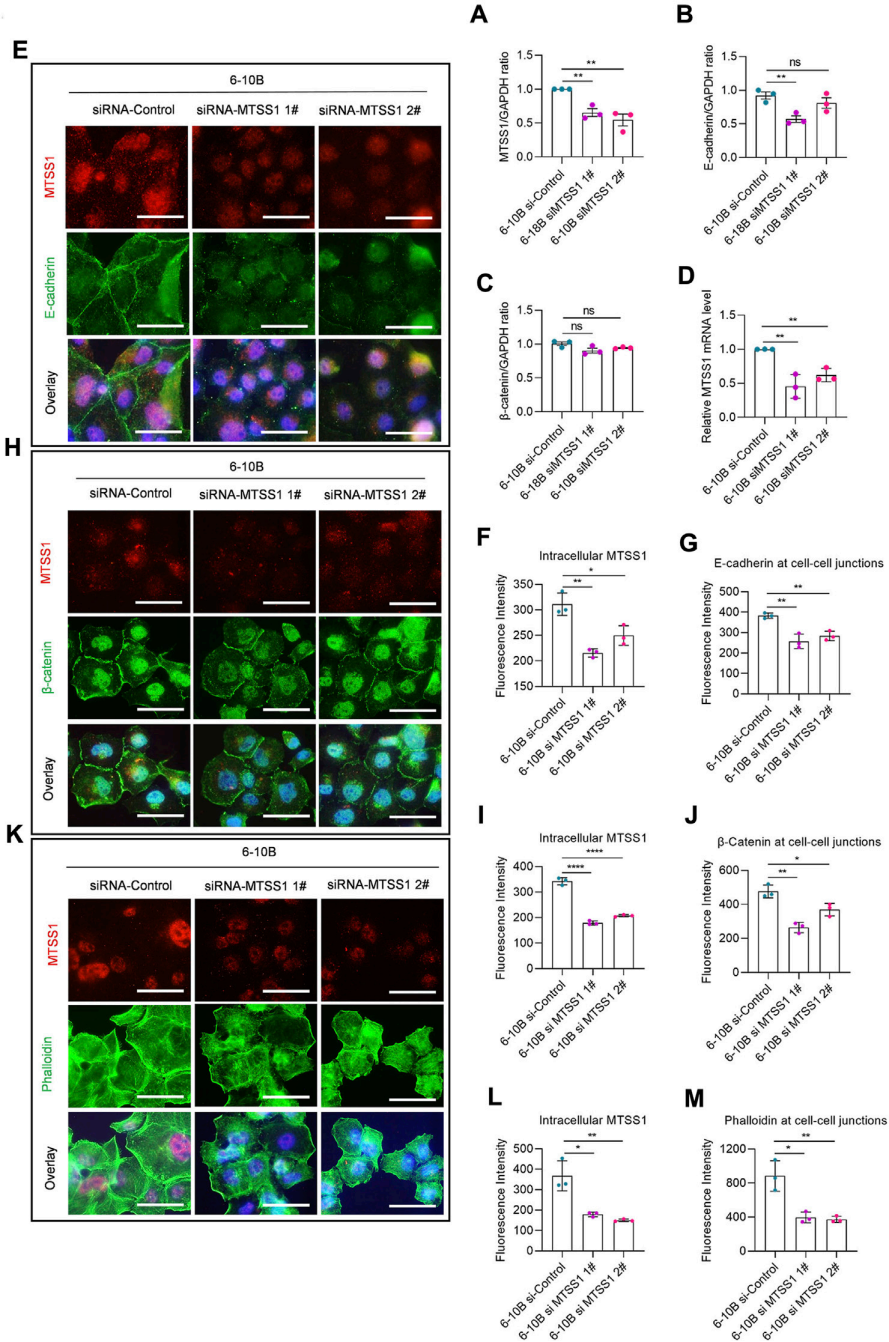
MTSS1 regulates actin dynamics for the formation of cell–cell junctions. **(A–C)** Relative expression level of MTSS1 **(A)**, E-cadherin **(B)** and β-catenin **(C)** by Western Blot. **(D)** Quantification of the efficiency of silencing with two different MTSS1-specific siRNAs by qPCR. **(E)** Representative immunofluorescence staining of adhering cells to show the adherens junctions. Red, TFP (tdTomato fluorescent protein)/MTSS1; green, E-cadherin; Blue, Hoechst. **(F, G)** Quantification of immunofluorescence intensity of intracellular MTSS1 **(F)** and E-cadherin at cell–cell junctions **(G)**, using the ZEN software. **(H)** Representative immunofluorescence staining of adhering cells to show the adherens junctions. Red, TFP (tdTomato fluorescent protein)/MTSS1; green, *β*-catenin; Blue, Hoechst. **(I, J)** Quantification of immunofluorescence intensity of intracellular MTSS1 **(I)** and *β*-catenin at cell–cell junctions **(J)**, using the ZEN software. **(K)** Representative immunofluorescence staining of adhering cells to show the cell morphology. Red, pCMV-tdTomato/MTSS1; green, phalloidin; Blue, Hoechst. Scale bar, 50 µm. **(L, M)** Quantification of immunofluorescence intensity of intracellular MTSS1 **(L)** and F-actin at cell–cell junctions **(M)**, using the ZEN software. Numerical data are presented as means ± SEM. *, *p* ≤ 0.05; **, *p* ≤ 0.01; ***, *p* ≤ 0.001; ****, *p* ≤ 0.0001; ns, *p* > 0.05 (n = 3 biological replicates/group; Unpaired, two-tailed Student’s *t*‐test).

Since increased expression of MTSS1 in cells with low levels of MTSS1 enhanced the adherens junctions, we analyzed if silencing of MTSS1 in NP69 and 6-10B cells resulted in a reciprocal phenotype, i.e., loss of cell adherens junctions. Successful silencing was shown by the reduced level of MTSS1 (Figure 5A; Figures 6A, D). In general, silencing of MTSS1 did not result in significant decreased expression of E-cadherin (Figure 6B) or *β*-catenin (Figure 6C). Compared to cells treated with control siRNA, cells treated with MTSS1-specific siRNA showed a reduced junctional localization of E-cadherin (Figures 6E–G) and *β*-catenin (Figures 6H–J), as well as junctional F-actin (Figures 6K–M) in 6-10B cells. A similar result was seen in NP 69 cells (Supplementary Figure S9), however, with the exception that these cells do not express E-cadherin.

## 4 Discussion

Functional studies at the cellular level have shown that MTSS1 has a major role as a scaffold protein in the regulation of actin dynamics (Machesky and Johnston, 2007). Overexpression of full-length MTSS1 leads to actin-based morphological reorganization, such as formation of filopodia (Woodings et al., 2003; Yamagishi et al., 2004; Gonzalez-Quevedo et al., 2005; Saarikangas et al., 2015), reshaping of lamellipodia and membrane ruffling, together with disassembly of stress fibers (Mattila et al., 2003; Woodings et al., 2003; Bompard et al., 2005; Gonzalez-Quevedo et al., 2005). We have confirmed previous findings that the expression of the MTSS1 I-BAR domain by itself is sufficient to induce filopodia formation (Yamagishi et al., 2004; Bompard et al., 2005). Indeed, in the present study, we showed that overexpression of the I-BAR domain of MTSS1 alone induced extensive alterations in filopodia and lamellipodia formation; conversely, inactivation of the I-BAR domain of MTSS1 resulted in formation of broad lamellipodia and loss of filopodia. This suggests that the intact I-BAR domain contributes to filopodia formation induced by full-length MTSS1 expression, while the other parts of MTSS1 that include a proline-rich region, a serine-rich region, and the WH2 domain, are involved in lamellipodia formation. Most likely, this is the reason why only the cells that expressed full-length MTSS1 showed balanced formation of filopodia and lamellipodia. It has been observed that the formation of filopodia-like protrusions is the early step in the formation of cell–cell junctions (Vasioukhin et al., 2000; Hoelzle and Svitkina, 2012). Mechanically, filopodia from 1 cell interdigitate across the middle line to the confronting cell membrane, forming adhesions at sites of membrane contact and then filopodia regress through actin to form mature adherens junctions (Wood and Martin, 2002).

Recently it has been reported that MTSS 1 acts as a tumor suppressor by inhibiting EMT in triple-negative breast cancer (Agarwal et al., 2017). However, using GSEA, we found that MTSS1 expression was negatively correlated with the process of EMT in the 5-8F cell line (Supplementary Figure S10A) while there was no correlation in the TW03 cell line (Supplementary Figure S10B). Of the common EMT genes, we only found E-cadherin CDHI to increase upon forced expression of MTSS1 in both 5-8F and TW03 NPC cells, compared to control cells. Taken together, MTSS1 seems to suppress tumor metastasis in NPC primarily by another mechanism than targeting EMT.

**FIGURE 7:**
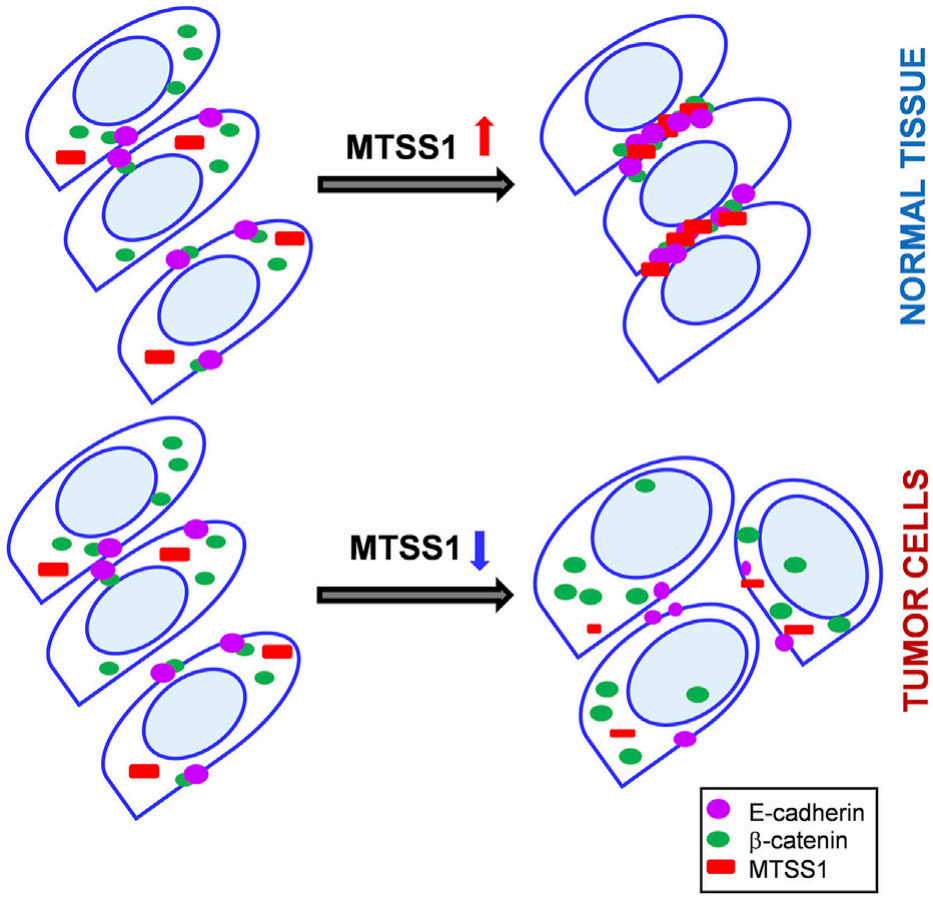
A schematic view on a role of MTSS1 in the control on an adherens junction formation. Top panel: Upon MTSS1 overexpression (red), interaction between E-cadherin (purple) and *β*-catenin (green) is promoted and tight adherens junctions are formed. Moreover, beta-catenin is retained in the cytoplasm. Bottom panel: MTSS1(red) is downregulated in cancerous cells, as a rule. *β*-catenin (green) is translocated to the nucleus and tumor-promoting genes are transactivated.

Altered expression of E-cadherin and *β*-catenin is frequent in cancers. *β*-catenin is a key player in the Wnt and E-cadherin signaling pathways, which are both involved in oncogenesis. Loss of E-cadherin results in *β*-catenin translocation to the nucleus, where it activates tumor-promoting genes (Gavert and Ben‐Ze’ev, 2007). Here, upon MTSS1 overexpression, E-cadherin accumulated at adherens junctions. While there was no increase in nuclear *β*-catenin or any activation of the Wnt/β-catenin signaling pathway (Supplementary Figure S5A); there was a significant accumulation of *β*-catenin in the cytosol at adherens junctions. This suggests that MTSS1 induces E-cadherin accumulation at cell junctions, where the E-cadherin binds to *β*-catenin, and thus prevents *β*-catenin translocation into the nucleus. In turn, increased *β*-catenin would facilitate E-cadherin stabilization at the cell–cell contact zone (Supplementary Figure S5B). Through its I-BAR domain, MTSS1 appears to be required for the formation of the functional E-cadherin–β-catenin complex at the cell–cell interface (Figure 7). Interestingly, MTSS1 affects the cell–cell junction complex through both the subcellular relocalization of proteins and the induction of expression of relevant genes, such as E-cadherin. It is not known which pathways are used to achieve this interesting and partially coordinated effect on the expression of this selected set of genes. Although there was no increase in nuclear *β*-catenin, it might be of relevance that four phosphatases regulated by MTSS1 are reported to dephosphorylate *β*-catenin, thereby regulating its subcellular localization (Yan et al., 2006; Zhou et al., 2016).

MTSS1 is a putative tumor suppressor in a variety of cancers, not only limited to epithelial cancers. Low expression of MTSS1 correlates with poor prognosis for patients with diffuse large B-cell lymphoma (Xu and Xu, 2018). In acute myeloid leukemia, MTSS1 is downregulated and the low expression of MTSS1 is associated with poor patient prognosis, chemotherapy resistance, and disease aggressiveness (Grandits et al., 2021). Moreover, treatment of acute promyelocytic leukemia cells with all-trans retinoic acid, which is included in the curative treatment regime, could increase MTSS1 mRNA levels (Schemionek et al., 2015). Similarly, in chronic myeloid leukemia, MTSS1 is downregulated, and re-expression of MTSS1 affects leukemic cell motility, tumor growth, and chronic myeloid leukemia development *in vitro* (Schemionek et al., 2016). Tyrosine kinase inhibitors increase MTSS1 mRNA levels (Schemionek et al., 2016), and MTSS1 levels were restored when patients had reached complete remission. Taken together, these data suggest that MTSS1 potentially can be targeted pharmacologically in the treatment of patients with acute myeloid leukemia and chronic myeloid leukemia, which of course could be of interest also for epithelial cancers with low MTSS1 expression.

Several mechanisms, including DNA-methylation, have been proposed to explain why MTSS1 is downregulated in cancers, and sometimes not at all expressed in metastases. Of note, the expression of MTSS1 is completely ablated in the C666-1 cell line, which is an EBV-positive NPC cell line expressing EBNA1 and LMP2A, which suggests that EBV might affect MTSS1 and enhance the downregulation.

In summary, downregulation of MTSS1 correlates with advanced tumor stages and poor prognosis in NPC. This is most likely related to the release of its suppressive role in tumor dissemination following MTSS1 downregulation. Our data suggest an important role for MTSS1 in the inhibition of the early steps in metastatic spread. Restoration of MTSS1 function will be an interesting therapeutic approach for prevention of metastasis formation in NPC, and in other cancers. For this purpose, it will be important to determine whether MTSS1 operates in a similar way to limit metastatic spread in other epithelial cancers.

The author(s) declare financial support was received for the research, authorship, and/or publication of this article.

## Data Availability

The datasets presented in this study can be found in online repositories. The names of the repository/repositories and accession number(s) can be found below: https://www.ncbi.nlm.nih.gov/geo/, GSE201394.
