# Supplementary material for: MTSS1 is downregulated in nasopharyngeal carcinoma (NPC) which disrupts adherens junctions leading to enhanced cell migration and invasion
Source: Front Cell Dev Biol. 2023 Oct 18;11:1275668. doi: 10.3389/fcell.2023.1275668 (PMC10618355; doi:10.3389/fcell.2023.1275668)
Supplement: Supplementary file 1 [file Table1.DOCX]

**Materials and Methods**

**Cell culture**

Seven EBV-negative NPC-derived cell lines were included in this study. The cells were cultured in Dulbecco's modified eagle medium (10566016; Gibco, New York, USA): 5-8F (RRID: CVCL_C528), 6-10B (RRID: CVCL_C529), TW03 (RRID: CVCL_6010), CNE1 (RRID: CVCL_6888), HONE1 (RRID: CVCL_8706), HK1 (RRID: CVCL_7084), and CNE2 (RRID: CVCL_6889) cells. One EBV-positive NPC cell line, C666-1 (RRID: CVCL_7949), was cultured in Roswell Park Memorial Institute 1640 (61870044; Gibco, New York, USA). Both of these culture media were supplemented with 10% fetal bovine serum (A4766801; Gibco, New York, USA) and penicillin–streptomycin (1000 U/mL; 1000 μg/mL, respectively; SV30010, HyClone). The immortalized human nasopharyngeal epithelial cell lines NP69 (RRID: CVCL_F755) and NP460 (RRID: CVCL_X205) were cultured in a 1:1 ratio of defined keratinocyte–serum-free medium (10744019; Gibco) and EpiLife medium (MEPI500CA; Gibco, New York, USA).

All of the cell lines were recently authenticated using STR profiling. Cells were maintained in a humidified incubator with an atmosphere of 5% CO_2_ at 37 °C. These cell lines were routinely treated with Plasmocin® (ant-mpt, InvivoGen, San Diego, USA) to eliminate mycoplasma infection. MycoStrip™ (rep-mys-10, InvivoGen, San Diego, USA) was used to detect mycoplasma contamination in cell cultures.

**Purification of plasmid DNA**

The vectors encoding Myc-tagged human MTSS1 FL (pRK5Myc-MTSS1; amino acids 1-759), MTSS1 K4D (pRK5Myc-MTSS1 K4D; mutant K149,150,152,153D), MTSS1 △I-BAR (pRK5Myc-MTSS1; amino acids 235-759), and MTSS1 I-BAR (pRK5Myc-MTSS1; amino acids 1-254) were kindly provided by Laura M. Machesky (Beatson Institute, Glasgow, UK) [1, 2]. The pCMV-tdTomato vector was purchased from Addgene (54642; Watertown, MA, USA) and the pCMV-Myc empty vector was from Clonetech (635689, Mountain View, CA, USA) The plasmids were propagated in *Escherichia coli* and purified using Qiagen kits (K210096; Hilden, Germany). These plasmids were verified by DNA sequencing by KIGene (Karolinska Institute, Stockholm, Sweden).

***In-vitro* DNA transfection**

The 5-8F and TW03 cells were plated the day before transfection to reach roughly 50% confluency on the day of transfection. The FuGENE HD transfection reagent (E2311; Promega, Madison, WI, USA) was mixed with plasmid DNA solution at a 3 μL:1 μg ratio and then diluted to the correct volume with reduced-serum medium (Opti-MEM; 31985047; Gibco, New York, USA), according to the manufacturer instructions. The diluted mixture was incubated for 10 min at room temperature before being added to the plates containing the cells. The cells were assayed 36 h after transfection.

***In vitro* small interfering RNA transfection**

Following the protocol of Lipofectamine RNAiMAX Reagent (13778; Invitrogen, Carlsbad, CA, USA), 6-10B and NP69 cells were seeded in a T25 flask and incubated for 16 hours to reach a 70% confluent monolayer of cells at the time of transfection. To prepare a siRNA–lipid complex 60 pmol predesigned MTSS1 small interfering RNA (siRNA) (AM16708, siRNA ID#s18915; Ambion, Austin, TX, USA) or non-targeting siRNA control (AM4390844; Ambion) and 20 μL Lipofectamine RNAiMAX Reagent were diluted in 300 μL Opti-MEM reduced serum medium, separately. The siRNA–lipid complex was incubated for 5 min at room temperature before being added to the cells. Cells were incubated for 48 h and then analyzed.

**RNA extraction, reverse transcription, and qPCR**

RNA was purified using the RNeasy mini kit (74106, Qiagen, Hilden, Germany) and cDNA was synthesized using the high-capacity cDNA reverse transcription kit with RNase inhibitor (4374967, Applied Biosystems, Waltham, MA, USA) according to the manufacturer’s protocols. qPCR was performed using a Real-time PCR instrument (96 wells; StepOnePlus, Applied Biosystems, Foster City, CA, USA) with a two-step PCR amplification using SYBR Green (A25742, Applied Biosystems, Waltham, MA, USA). Relative gene expression levels for each gene set were normalized to GAPDH or β-actin and compared using 2^−ΔΔCT^ method. The q-PCR primers are listed in Supplementary Table 1.

**Immunoblotting**

Protein lysates were prepared and analyzed by a conventional western blot (WB) assay as described previously [3]. Protein content in cell lysates was measured by BCA Protein Assay Kit（23250, ThermoFisher Scientific,USA）relative to a mitochondrial protein. 30 μg/well was loaded on a SDS gel. Signals from enhanced chemiluminiscence reagent (ECL, Amersham, Piscataway, NJ, USA), used in the WB assay, were acquired by a BIORAD analyzer/a ChemiDoc XRS+(Bio-Rad Laboratories, Hercules, CA, USA) with IMAGE LAB^TM^software (Bio-Rad Laboratories, Hercules, CA, USA).

**Meta-analysis of microarray datasets**

A comprehensive search for studies on NPC was carried out in the Gene Expression Omnibus (GEO) datasets, to identify data on MTSS1 expression in NPC tissue and normal epithelium. Seven publicly available datasets, based on expression array profiling, were included in a meta-analysis. The raw data was analyzed with GEO2R, an interactive web tool used to identify genes that are differentially expressed across different groups. It includes intrinsic normalization based on the whole test set. Excel was used to collect the data of MTSS1 expression in each array for subsequent meta-analysis. The MTSS1 expression in these seven microarrays was pooled using the Review Manager 5.4 software (desktop), applying the inverse variance method of random models. Standard mean difference was used to represent the differences between the microarrays in this platform application.

**Wound healing assay**

Cells (60,000 cells per well) were seeded on Ibidi Culture-Insert 2 Well plates (80209, Gräfelfing, Germany) and left overnight to reach a confluent cell monolayer. Thereafter, the inserts were removed to create cell-free gaps. The widths of the gaps were photographed at 0 h and at 14 h after removal of the inserts using a microscope with a camera (IX73; Olympus Life Science, Shinjuku, Tokyo, Japan). Quantification of the wound healing assays was performed using the ImageJ software (NIH). The transfection efficiency was measured by counting cells expressing MTSS1 or td-Tomato. The transfection efficiency was approximately 50% in td-Tomato group and 35% in the MTSS1 group (data not shown).

**Cell migration and invasion assays**

Cells (1 ×10^5^ per insert) were seeded into Transwell inserts with a polyethylene terephthalate (PET) membrane (pore size, 8 μm; Thermo Fisher Scientific) in 24‐well plates with 15% fetal bovine serum as chemo-attractant. After 24 h, the medium within the Transwell inserts was carefully removed. The cells were then fixed with 4% formaldehyde for 10 min, permeabilized with 0.01% Triton X‐100 (Sigma‐Aldrich, St. Louis, MO, USA), and stained with crystal violet (HT90132; Sigma‐Aldrich, St. Louis, MO, USA). Cells that did not migrate across the Transwell membrane were then removed by gently wiping with a cotton swab. The migrated cells were photographed under the phase‐contrast microscope (IX73; Olympus Life Science, Shinjuku, Tokyo, Japan). A similar protocol was used for the invasion assay but the cells were instead seeded on Matrigel invasion chambers (BioCoat Matrigel with 8.0 µm PET membrane, 354480, Corning, New York, USA).

**Immunofluorescence staining**

The 5-8F and TW03 cells were fixed 36 h after transfected in 4% formaldehyde for 10 min at 37 °C, permeabilized with 0.1% Triton X-100 for 10 min, or with ice-cold pure methanol for 2.5 min, and blocked in 5% bovine serum albumin in phosphate-buffered saline (PBS) for 1 h at room temperature. The primary (dilution1:100) and secondary antibodies (dilution 1:1000) were diluted in PBS and were added for overnight incubation at 4 °C and for 1 h at room temperature, respectively. F-actin was visualized using Alexa Fluor 488-conjugated phalloidin (A12379; Invitrogen, Carlsbad, CA, USA) diluted at 1:200. Before mounting, nuclei were stained with Hoechst 33342 (62249; Thermo Scientific, Waltham, MA, USA) at room temperature for 10 min. Finally, the cells were examined and photographed using a microscope (Axiovert 200M; Carl Zeiss AG, Oberkochen, Germany) with a 63X immersion oil objective (numeric aperture 1.4). The data were analyzed with the ZEN (blue edition) 3.1 software (Zeiss). The total number of transfected cells analysed to quantify the area of lamellipodia, the length of filopodia were all 40-50. The experiments were repeated five times.

**RNA sequencing and gene-set enrichment analysis**

Total RNA was subjected to quality control with Agilent TapeStation according to the manufacturer’s instructions. To construct libraries suitable for Illumina sequencing, the Illumina Stranded mRNA Prep, Ligation preparation protocol was used which includes mRNA isolation, cDNA synthesis, ligation of anchors and amplification and indexing of the libraries. The yield and quality of the amplified libraries were analyzed using Qubit by Thermo Fisher and the Agilent TapeStation. The indexed cDNA libraries were normalized and combined, and the pools were sequenced on the Nextseq 2000, P2 100 cycle kit (20046811, Illumina, CA, USA), paired-end mode (Read 1: 52 cycles, Read 2: 52 cycles, index 1: 10 cycles, Index 2: 10 cycles). Downstream analysis was performed using a combination of programs, which included TapeStation Analysis Software A.02.02 (SR1), STAR, HTseq, Cufflink, and wrapped scripts. Alignments were analyzed using the Tophat program, and differential expression was determined using DESeq2, with Wald tests.

Comparisons of the gene expression between the Myc-tagged empty vector (pCMV-Myc) and Myc-MTSS1 (pRK5Myc-MTSS1 FL) samples were carried out to determine the differentially regulated genes. A few different approaches were used for this analysis. We used the general linear model approach with the Voom approach, where samples and genes are assigned weights and then modeled using linear models. Also, the genes were filtered to remove non/low expressed genes. Filtering was carried out by keeping genes that had one count per million in three or more samples (cpm1_3). Genes are considered to be deregulated if there was a > two-fold difference of up- or downregulation with an adjusted *p*-value was < 0.05 for multiple hypothesis testing. Gene set enrichment analysis (GSEA) was performed using the GSEA software (Broad Institute, RRID: SCR_003199).

**Human specimens**

The inclusion criteria for this study were histological diagnostic of certainty for NPC (NPC group) or normal nasopharyngeal epithelium (NNE group). Patients with the diagnosis of recurrent NPC will be excluded. A tissue microarray containing 131 cases of primary NPC with clinically relevant information was purchased from Outdo Biotech (HNasN132Su01; Shanghai, China). Normal nasopharyngeal epithelium was derived from patients with suspect NPC who were confirmed not to have NPC upon histopathological examination. The normal nasopharyngeal epithelium and primary NPC biopsies were obtained from the Department of Otolaryngology-Head and Neck Surgery, First Affiliated Hospital of Guangxi Medical University (Nanning, Guangxi, China). The specimen collection was approved by the Research Ethics Committee of First Affiliated Hospital of Guangxi Medical University (Ref. N° 2016-175, Ref. N° 2021-163) and Regionala Etikprövningsnämnden, Stockholm (Ref. N° 00-312). The specimen collection was undertaken with the understanding and written consent of each subject, and the specimens were anonymized. The study methodologies conformed to the standards set by the Declaration of Helsinki.

**Immunohistochemistry staining and scoring**

Paraffin sections were baked overnight at 50 °C. Deparaffinization and rehydration were performed in xylene and graded ethanol to distilled water. The sections then went through heat-induced epitope retrieval in citrate buffer (0.01 M, pH 6.0) using a pressure boiler as the heat source, for 20 min at 100 °C. Ultra V Block (TA-125-UB; Thermo Scientific) was used to reduce nonspecific binding, for 30 min at room temperature. The primary anti-MTSS1 antibody (HPA075540; RRID: AB_2686756) was applied to the samples overnight at 4 °C. The samples were then incubated with goat anti-rabbit IgG (H+L)-cross-adsorbed horseradish peroxidase conjugate (A16104SAMPLE; Invitrogen, Carlsbad, CA, USA) for 30 min at room temperature, and developed in DAB solution (TA-999-QHDX; Thermo Scientific) for 5 min. Finally, the sections were counterstained with hematoxylin for 5 min. As negative controls, NPC sections were incubated with isotype-matched IgG. The stained sections were photographed using a microscope (IX73; Olympus Life Science, Shinjuku, Tokyo, Japan). Three fields of each section were scored blindly, as follows: immunohistochemistry (IHC) score = mean stain intensity (0-3) × percentage positive stain (0, <10%; 1, 10%-25%; 2, 25%-50%; 3, 50%-74%; 4, >75%). The representative images of IHC staining of MTSS1 in NPC biopsies were shown in Supplementary Fig.1.

**Statistical analysis**

We performed Meta-analysis of microarray data using Review Manager 5.4 software, and all the other analyses were conducted using GraphPad PRISM version 9.0 (GraphPad Software, San Diego, CA, USA). Three biological replicates were prepared for each group in all *in vitro* experiments. Numerical data were presented as means ± standard error of mean (SEM) and an unpaired Student's *t*‐test was utilized for comparison between two groups. However, Mann Whitney U test (Wilcoxon rank-sum test) was used to compare the expression of MTSS1 in NPC and NNE samples due to non-normal distribution of the data. A two-sided *P* value less than 0.05 was considered statistically significant. The IHC score of MTSS1 expression in NPC tissue sections was categorized into three groups by tertile, i.e., high (n=44), medium (n=43), and low (n=44) to evaluate the association between MTSS1 expression and the prognosis of NPC (Supplementary Figure 1). The overall survival time was defined as the time between the date of diagnosis and the date of death by any cause. The Kaplan-Meier method [4] was used for overall survival analysis, and the log-rank test was applied to assess the difference between groups. The receiver operating characteristic curve analysis[5] was used to investigate the predictive value of MTSS1 expression for the prognosis of NPC patients.

1. Bompard, G., Sharp, S.J., Freiss, G. & Machesky, L.M. Involvement of Rac in actin cytoskeleton rearrangements induced by MIM-B. *Journal of cell science* **118**, 5393-5403 (2005).
2. Dawson, J.C., Bruche, S., Spence, H.J., Braga, V.M. & Machesky, L.M. Mtss1 promotes cell-cell junction assembly and stability through the small GTPase Rac1. *PLoS One* **7**, e31141 (2012).
3. Liang J, Zheng S, Xiao X, Wei J, Zhang Z, Ernberg I, Matskova L, Huang G, Zhou X. Epstein-Barr virus-encoded LMP2A stimulates migration of nasopharyngeal carcinoma cells via the EGFR/Ca2+/calpain/ITGβ4 axis. Biol Open. 2017 Jun 15;6(6):914-922. doi: 10.1242/bio.024646. PMID: 28512118; PMCID: PMC5483025.
4. Dennis G, Jr., Sherman BT, Hosack DA, Yang J, Gao W, Lane HC & Lempicki RA (2003) DAVID: Database for Annotation, Visualization, and Integrated Discovery. Genome biology 4, P3.
5. Søreide K (2009) Receiver-operating characteristic curve analysis in diagnostic, prognostic and predictive biomarker research. Journal of clinical pathology 62, 1-5, doi: 10.1136/jcp.2008.061010.
