# Supplementary material for: MTSS1 is downregulated in nasopharyngeal carcinoma (NPC) which disrupts adherens junctions leading to enhanced cell migration and invasion
Source: Front Cell Dev Biol. 2023 Oct 18;11:1275668. doi: 10.3389/fcell.2023.1275668 (PMC10618355; doi:10.3389/fcell.2023.1275668)
Supplement: Supplementary file 2 [file Table2.docx]

**Supplementary Figure 1. MTSS1 is down-regulated in nasopharyngeal carcinoma (NPC).**

(**a**) Workflow of integrative gene expression meta-analysis from the Gene Expression Omnibus (GEO) database. (**b**) Meta-analysis of the GEO datasets to evaluate integrative MTSS1 expression. Total=number of tissues analyzed. IV, Intervention. CI, Confidence interval. (**c**) Workflow of clinicopathological analysis of MTSS1 expression in NPC tissue. (**d**) RT-qPCR analysis of relative MTSS1 expression in 5-8F and TW03 NPC cell lines to assess the transfection efficiency. ****, p ≤0.0001 (n = three biological replicates/group; Unpaired, two-tailed Student's t‐tests).

**Supplementary Figure 2. Integration of high-throughput RNA-Seq gene expression data and proteomic data suggest that MTSS1 induces adherens junction assembly.**

(**a-f**) Gene set enrichment analysis enrichment plots of the reactome cell junction organization (**a**), reactome adherens junction interaction (**b**), Gene Ontology molecular function (GOMF) actin monomer binding (**c**) and GOMF cadherin binding involved in cell–cell adhesion (**d**), Gene Ontology cellular component (GOCC) adherens junction (**e**), and (**f**). GOCC catenin complex. (**g**) Genes contributing most to the enrichment signal of a given gene set. *, *p* ≤0.05, ***, *p* ≤0.001, ****, *p* ≤0.0001 (n = three biological replicates/group; Unpaired, two-tailed Student's *t*‐tests).

**Supplementary Figure 3. MTSS1 does not play a major role in the organization of focal junctions, tight junctions and desmosomes.**

(**a**) RT–qPCR analysis of expression of genes involved in cell–cell junction assembly. (**b**) Representative images for immunofluorescence staining of markers involved in cell–cell junction organization. Red, TFP (tdTomato fluorescent protein)/MTSS1; green, JAM1/Claudin1/DSP/ ITGβ4; Blue, Hoechst. Scale bar, 50 µm. Numerical data are presented as means ± SEM. *, *P* ≤0.05; **, *P* ≤0.01; ns, *P* >0.05 (n = 3 biological replicates/group; Unpaired, two-tailed Student's *t*‐test).

**Supplementary Figure 4. The I-BAR domain is sufficient for MTSS1 to promote the formation of E-cadherin/β-catenin mediated cell adherens junction.**

(**a**) Representative immunofluorescence staining of adhering TW03 NPC cells. Red, TFP (tdTomato fluorescent protein)/MTSS1; green, E-cadherin; Blue, Hoechst. Scale bar, 50 µm. (**b**) Quantification of immunofluorescence intensity of E-cadherin at adherens junctions, using the ZEN software. (**c**) Representative immunofluorescence staining of adhering TW03 NPC cells. Red, TFP (tdTomato fluorescent protein)/MTSS1; green, β-catenin; Blue, Hoechst. Scale bar = 50 µm. (**d, e**) Quantification of immunofluorescence intensity of β-catenin at adherens junctions (**d**) and nucleus (**e**), using the ZEN software. (**f**) Percentage of red positive cells among the total cells. Numerical data are presented as means ± SEM. *, *P* ≤0.05; **, *P* ≤0.01; ***, *P* ≤0.001; ns, *P* >0.05 (n = 3 biological replicates/group; Unpaired, two-tailed Student's *t*‐test).

**Supplementary Figure 5. MTSS1 does not active Wnt/ β-catenin signaling and MTSS1 stabilizes E-cadherin in NPC cells.**

(**a**) Gene set enrichment analysis enrichment plots of Wnt/ β-catenin signaling. (**b**) Gene set enrichment analysis enrichment plots of E-cadherin stabilization pathway. (**c**) Fold change of genes involved in dephosphorylation of beta-catenin in MTSS1 positive 5-8F and TW03 cells. PTPRU, protein tyrosine phosphatase receptor type U; PTPRF, protein tyrosine phosphatase receptor type F; PTPRG, protein tyrosine phosphatase receptor type G; PTPRK, protein tyrosine phosphatase receptor type K.

**Supplementary Figure 6. MTSS1 regulates actin dynamics for the formation of cell–cell junctions.** (**a**) Representative immunofluorescence staining of adhering TW03 NPC cells to show the cell morphology. Red, TFP (tdTomato fluorescent protein)/MTSS1; green, phalloidin; Blue, Hoechst. Scale bar, 50 µm. (**b-d**) Quantification of filopodia length (**b**) and lamellipodia area (**c**) along the cell edge, and cell polarity (**d**). (**e**) Summary of the TW03 NPC cell morphology. (**f**) Representative immunofluorescence staining of adhering TW03 NPC cells to show the adherens junctions. Red, TFP (tdTomato fluorescent protein)/MTSS1; green, phalloidin; Blue, Hoechst. Scale bar, 50 µm. (**g**) Quantification of immunofluorescence intensity of F-actin at cell–cell junctions, using the ZEN software. Numerical data are presented as means ± SEM. *, *P* ≤0.05; **, *P* ≤0.01; ***, *P* ≤0.001; ns, *P* >0.05 (n = 3 biological replicates/group; Unpaired, two-tailed Student's *t*‐test).

**Supplementary Figure 7.** **MTSS1 control of cell migration depends on its I-BAR domain.**

(**a**) Representative images of cell migration assay in TW03 cells. Scale bar, 250 µm. (**b**) Representative images of cell invasion assay. Scale bar, 250 µm. (**c**) Quantification of cell migration. (**d**) Quantification of cell invasion. (**e**) Representative microscopy images of wound closure of cells at 0 h and 14 h after creating a cell‐free zone. Red, TFP (tdTomato fluorescent protein)/ MTSS1; Blue, Hoechst. Scale bar, 500 µm. (**f**) Quantification of gap closure. Numerical data are presented as means ± SEM. *, *P* ≤0.05; **, *P* ≤0.01; ***, *P* ≤0.001; ****, *P* ≤0.0001; ns, *P* >0.05 (n = 3 biological replicates/group; Unpaired, two-tailed Student's *t*‐test).

**Supplementary Figure 8.** **MTSS1 control of cell migration depends on its I-BAR domain.**

(**a**) Representative images of cell migration assay in 5-8F cells. Scale bar, 250 µm. (**b**) Representative images of cell invasion assay. Scale bar, 250 µm. (**c**) Quantification of cell migration. (**d**) Quantification of cell invasion. (**e**) Representative microscopy images of wound closure of cells at 0 h and 14 h after creating a cell‐free zone. Red, TFP (tdTomato fluorescent protein)/ MTSS1; Blue, Hoechst. Scale bar, 500 µm. (**f**) Quantification of gap closure. Numerical data are presented as means ± SEM. *, *P* ≤0.05; **, *P* ≤0.01; ***, *P* ≤0.001; ****, *P* ≤0.0001; ns, *P* >0.05 (n = 3 biological replicates/group; Unpaired, two-tailed Student's *t*‐test).

**Supplementary Figure 9. MTSS1 regulates actin dynamics for the formation of cell–cell junctions.** (**a, b**) Relative expression level of MTSS1 (**a**) and β-catenin (**b**) by Western blotting. (**c**) Quantification of the efficiency of silencing with two different MTSS1-specific siRNAs by qPCR. (**d**) Representative immunofluorescence staining of adhering cells to show the adherens junctions. Red, TFP (tdTomato fluorescent protein)/MTSS1; green, β-catenin; Blue, Hoechst. Scale bar, 50 µm. (**e,f**) Quantification of immunofluorescence intensity of intracellular MTSS1 (**e**) and β-catenin at cell–cell junctions (**f**), using the ZEN software. (**g**) Representative immunofluorescence staining of adhering cells to show the cell morphology. Red, TFP (tdTomato fluorescent protein)/MTSS1; green, phalloidin; Blue, Hoechst. Scale bar, 50 µm. (**h**, **i**) Quantification of immunofluorescence intensity of intracellular MTSS1 (**h**) and F-actin at cell–cell junctions (**i**), using the ZEN software. Numerical data are presented as means ± SEM. *, *P* ≤0.05; **, *P* ≤0.01; ***, *P* ≤0.001; ****, *P* ≤0.0001; ns, *P* >0.05 (n = 3 biological replicates/group; Unpaired, two-tailed Student's *t*‐test).

**Supplementary Figure 10. MTSS1 suppresses NPC metastasis independent of EMT.**

(**a**) Gene set enrichment analysis enrichment plots of EMT. (**b**) RT–qPCR analysis of expression of genes involved in EMT. Numerical data are given as means ± SEM. *, *P* ≤0.05; ***, *P* ≤0.001; ns, *P* >0.05 (n = 3 biological replicates/group; Unpaired, two-tailed Student's *t*‐test).

**Supplementary Figure 11. Original Western blots.** Original Western blots used in Fig.3g.

**Supplementary Figure 12. Original Western blots.** Original Western blots used in Fig.5a.

**Supplementary Figure 13. Original Western blots.** Original Western blots used in Fig.5a.
