# Supplementary material for: MTSS1 is downregulated in nasopharyngeal carcinoma (NPC) which disrupts adherens junctions leading to enhanced cell migration and invasion
Source: Front Cell Dev Biol. 2023 Oct 18;11:1275668. doi: 10.3389/fcell.2023.1275668 (PMC10618355; doi:10.3389/fcell.2023.1275668)
Supplement: Supplementary file 3 [file DataSheet1.PDF]

Supplementary Fig.1

a

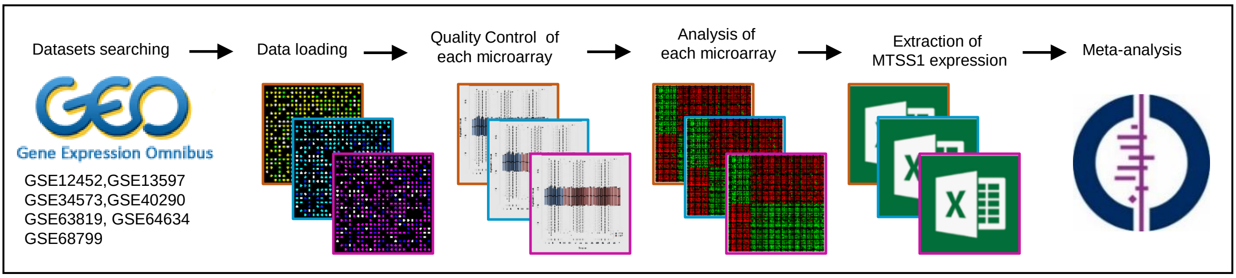

b

Study or Subgroup	NPC Mean	NPC SD	NPC Total	NNE Mean	NNE SD	NNE Total	Weight	Std. Mean Difference IV, Random, 95% CI
GSE12452	9.44	1.13	31	10.85	0.4	10	18.2%	-1.37 [-2.15, -0.59]
GSE13597	6.05	2.52	25	7.05	1.7	3	10.2%	-0.39 [-1.60, 0.81]
GSE34573	9.76	10.96	15	12.98	3.9	3	9.7%	-0.30 [-1.54, 0.95]
GSE40290	1.38	0.58	25	1.98	0.63	8	16.8%	-0.99 [-1.83, -0.15]
GSE53819	11.28	1.14	18	11.64	0.39	18	21.6%	-0.41 [-1.07, 0.25]
GSE64634	7.92	1.25	12	8.76	0.47	4	10.7%	-0.70 [-1.87, 0.46]
GSE68799	8.87	8.09	42	5.23	3.96	4	12.8%	0.45 [-0.58, 1.48]
**Total (95% CI)**			**168**			**50**	**100.0%**	**-0.59 [-1.03, -0.15]**

Heterogeneity: $Tau^2 = 0.12$; $Chi^2 = 9.28$, df = 6 (P = 0.16); $I^2 = 35\%$
Test for overall effect: Z = 2.61 (P = 0.009)

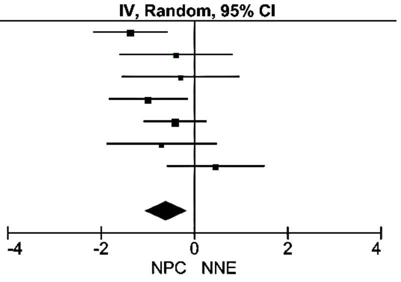

c

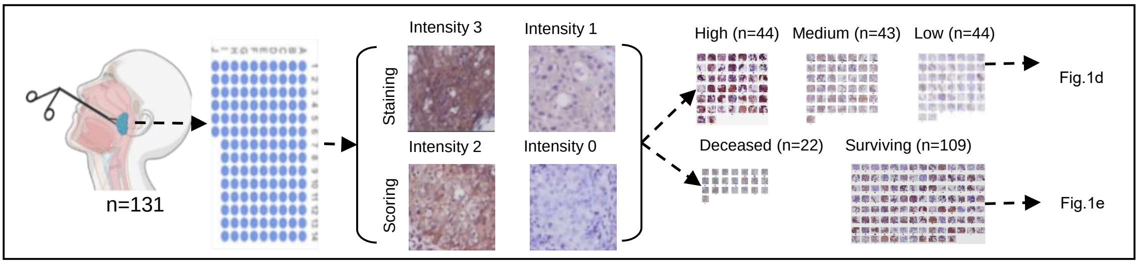

d

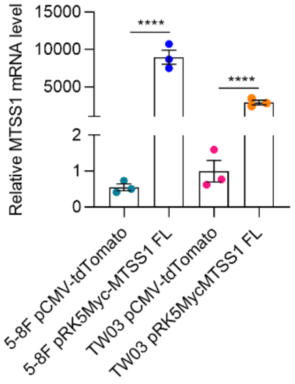

Supplementary Fig.2

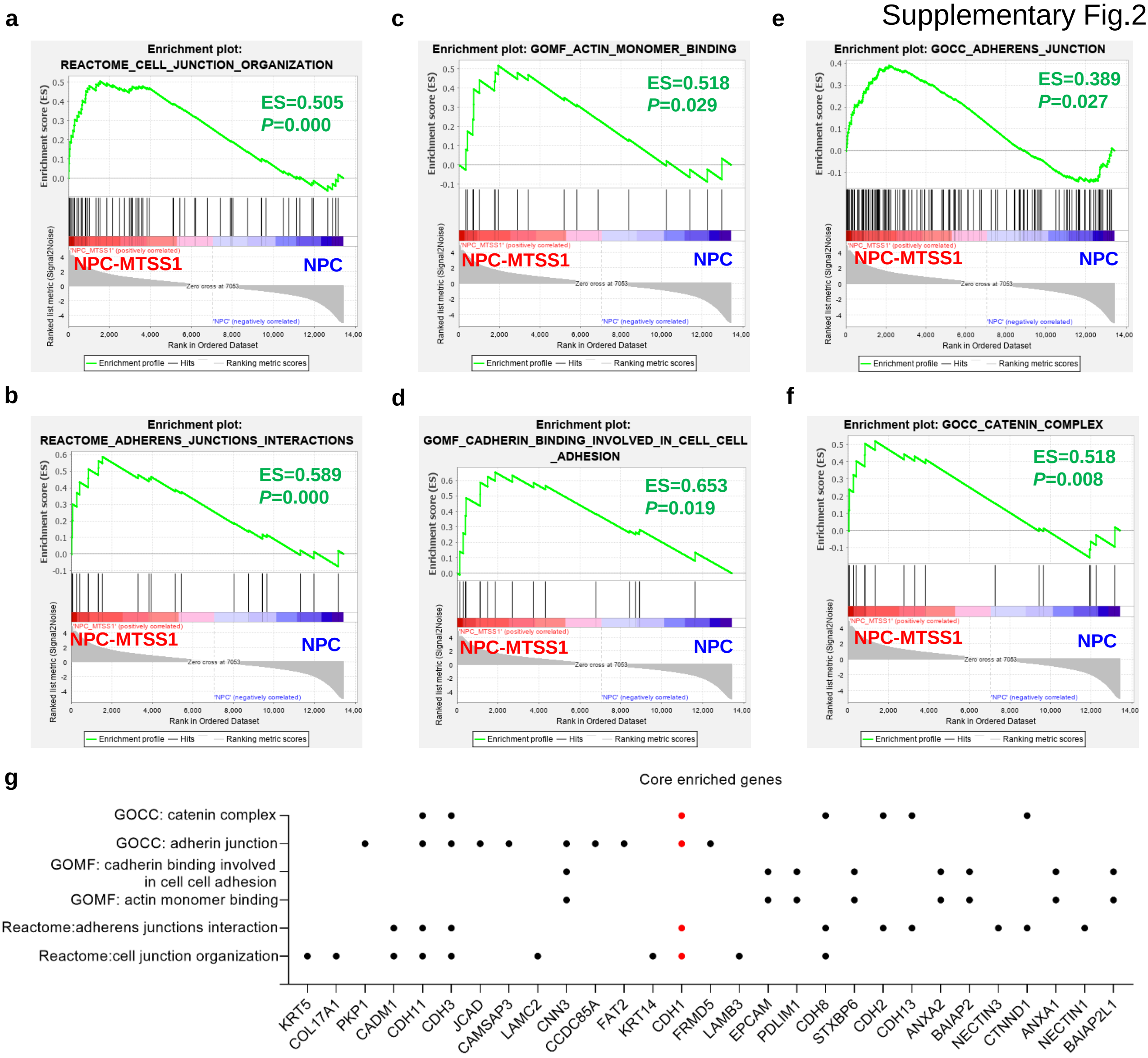

a

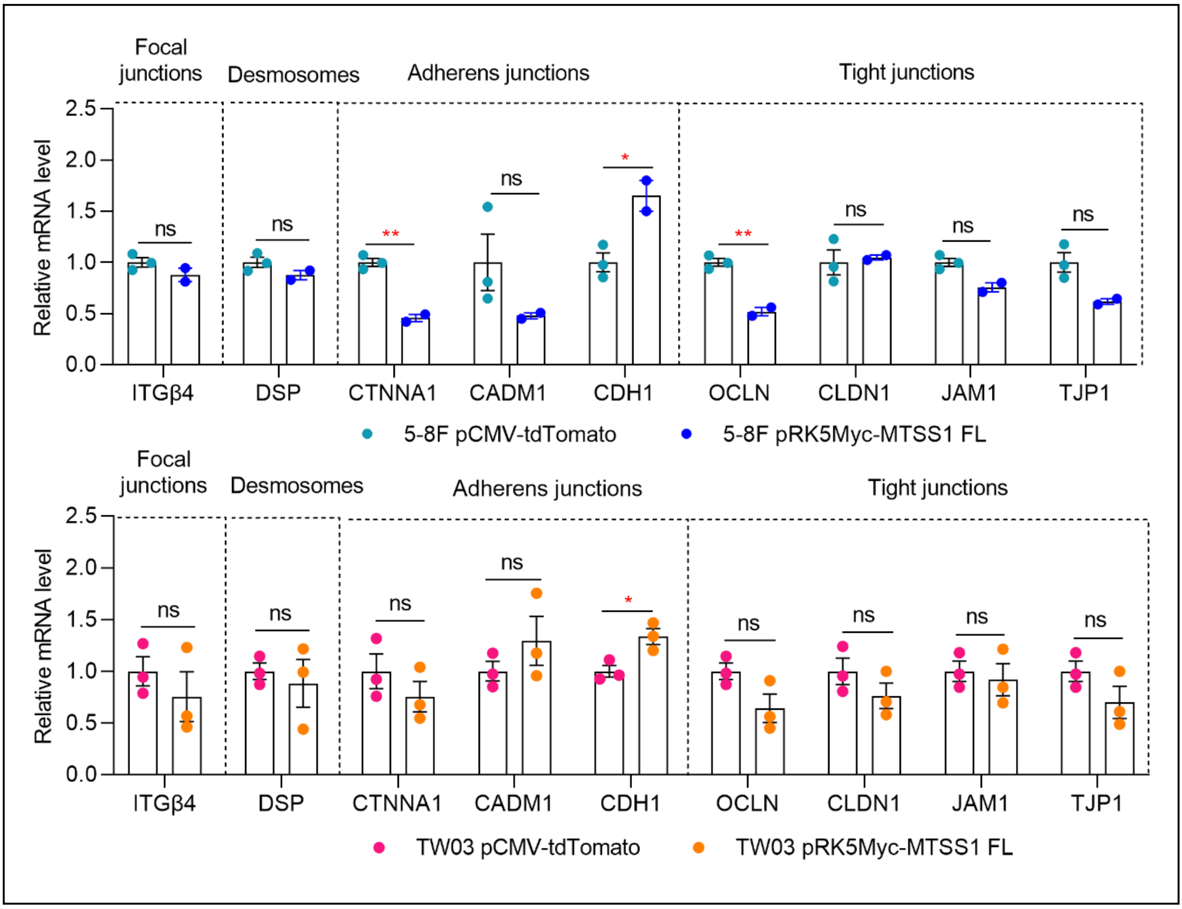
Focal junctions
Desmosomes
Adherens junctions
Tight junctions
Relative mRNA level
ns
**
*
ITGβ4
DSP
CTNNA1
CADM1
CDH1
OCLN
CLDN1
JAM1
TJP1
5-8F pCMV-tdTomato
5-8F pRK5Myc-MTSS1 FL
TW03 pCMV-tdTomato
TW03 pRK5Myc-MTSS1 FL

b

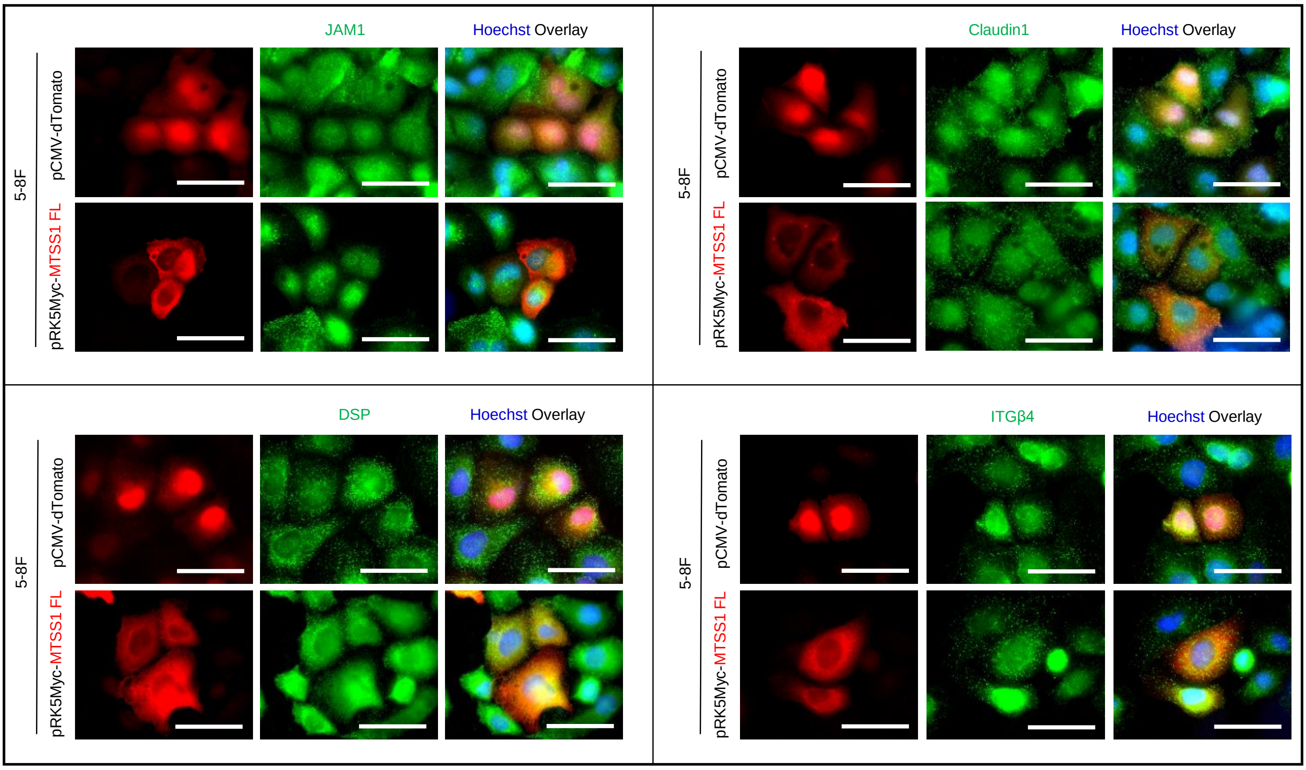
JAM1
Hoechst Overlay
Claudin1
DSP
ITGβ4
5-8F
pCMV-dTomato
pRK5Myc-MTSS1 FL

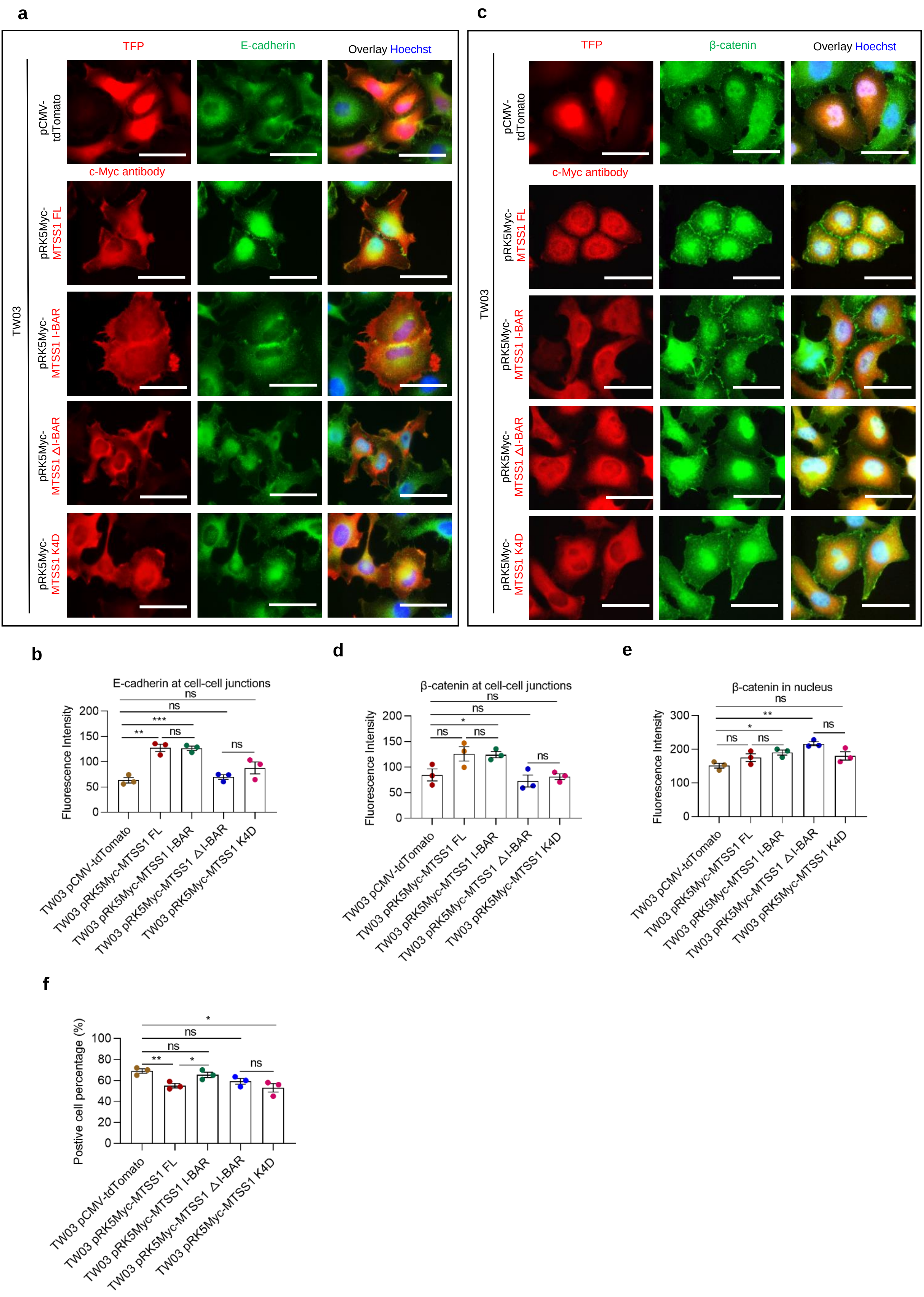
a
TFP
E-cadherin
Overlay Hoechst
c-Myc antibody
TW03
pCMV-tdTomato
pRK5Myc-MTSS1 FL
pRK5Myc-MTSS1 I-BAR
pRK5Myc-MTSS1 ΔI-BAR
pRK5Myc-MTSS1 K4D
c
TFP
β-catenin
Overlay Hoechst
c-Myc antibody
TW03
pCMV-tdTomato
pRK5Myc-MTSS1 FL
pRK5Myc-MTSS1 I-BAR
pRK5Myc-MTSS1 ΔI-BAR
pRK5Myc-MTSS1 K4D
b
E-cadherin at cell-cell junctions
Fluorescence Intensity
ns
***
**
d
β-catenin at cell-cell junctions
Fluorescence Intensity
ns
*
e
β-catenin in nucleus
Fluorescence Intensity
ns
**
*
f
Postive cell percentage (%)
*
ns
**
TW03 pCMV-tdTomato
TW03 pRK5Myc-MTSS1 FL
TW03 pRK5Myc-MTSS1 I-BAR
TW03 pRK5Myc-MTSS1 ΔI-BAR
TW03 pRK5Myc-MTSS1 K4D

a

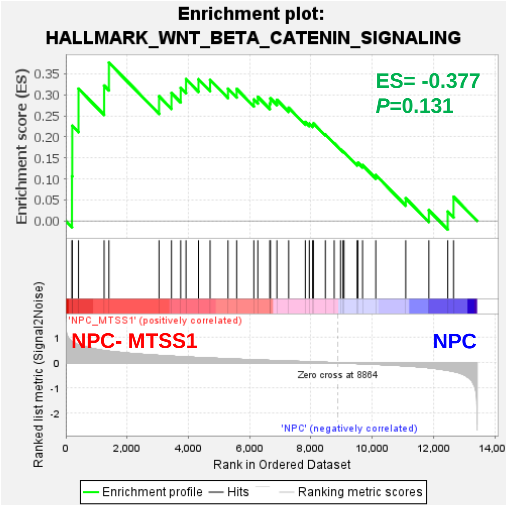
Enrichment plot:
HALLMARK_WNT_BETA_CATENIN_SIGNALING
ES= -0.377
P=0.131
Enrichment score (ES)
NPC- MTSS1
NPC
'NPC_MTSS1' (positively correlated)
Zero cross at 8864
'NPC' (negatively correlated)
Ranked list metric (Signal2Noise)
Rank in Ordered Dataset
Enrichment profile
Hits
Ranking metric scores

b

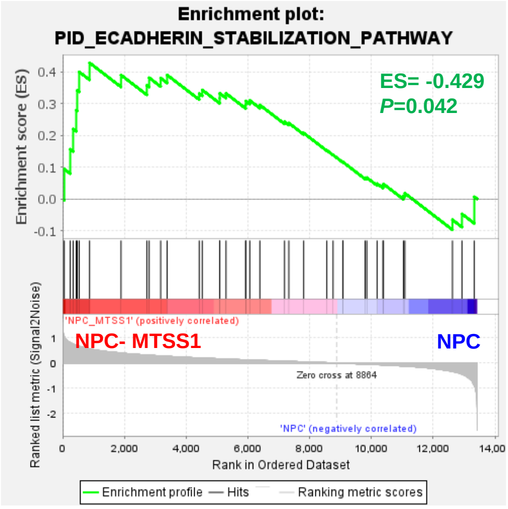
Enrichment plot:
PID_ECADHERIN_STABILIZATION_PATHWAY
ES= -0.429
P=0.042
Enrichment score (ES)
NPC- MTSS1
NPC
'NPC_MTSS1' (positively correlated)
Zero cross at 8864
'NPC' (negatively correlated)
Ranked list metric (Signal2Noise)
Rank in Ordered Dataset
Enrichment profile
Hits
Ranking metric scores

c

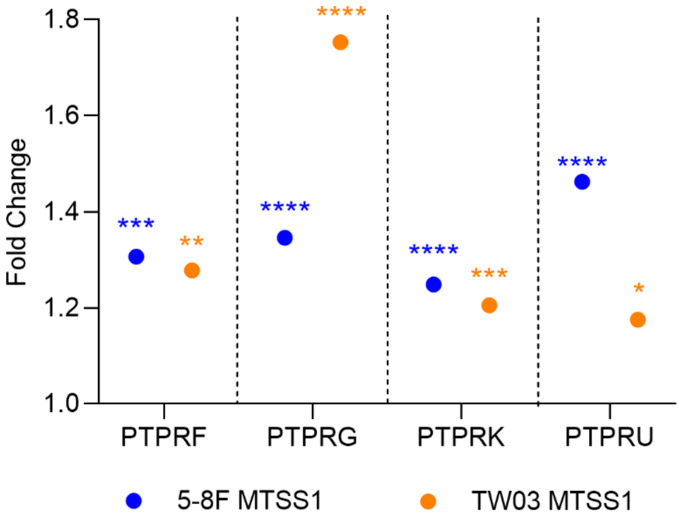
Fold Change
1.8
1.6
1.4
1.2
1.0
PTPRF
PTPRG
PTPRK
PTPRU
5-8F MTSS1
TW03 MTSS1

Supplementary Fig.6

a f

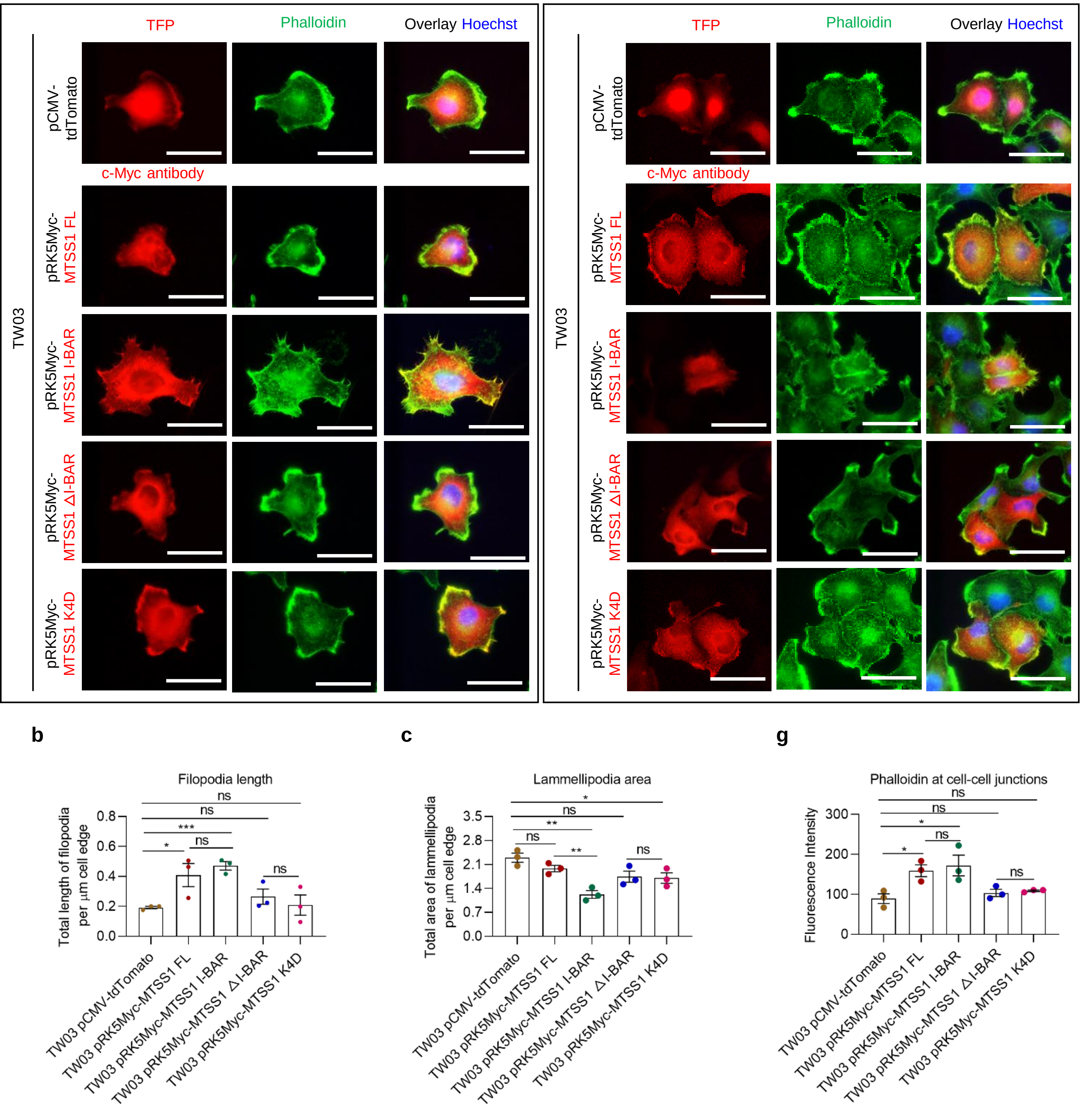

d

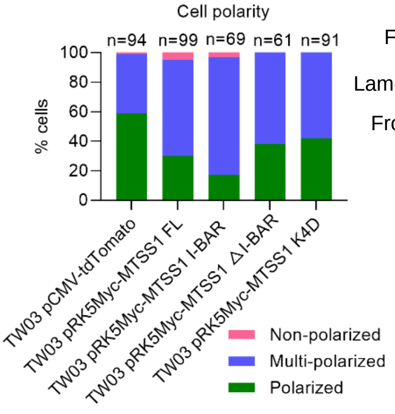

e

	TW03 pCMV-tdTomato	TW03 pRK5Myc-MTSS1 FL	TW03 pRK5Myc-MTSS1 I-BAR	TW03 pRK5Myc-MTSS1 △ I-BAR	TW03 pRK5Myc-MTSS1 K4D
Filopodia	+	++	++++	+	+
Lamellipodia	++++	++++	++	+++	++++
Front-rear polarity	++++	++	+	+++	+++

Supplementary Fig.7

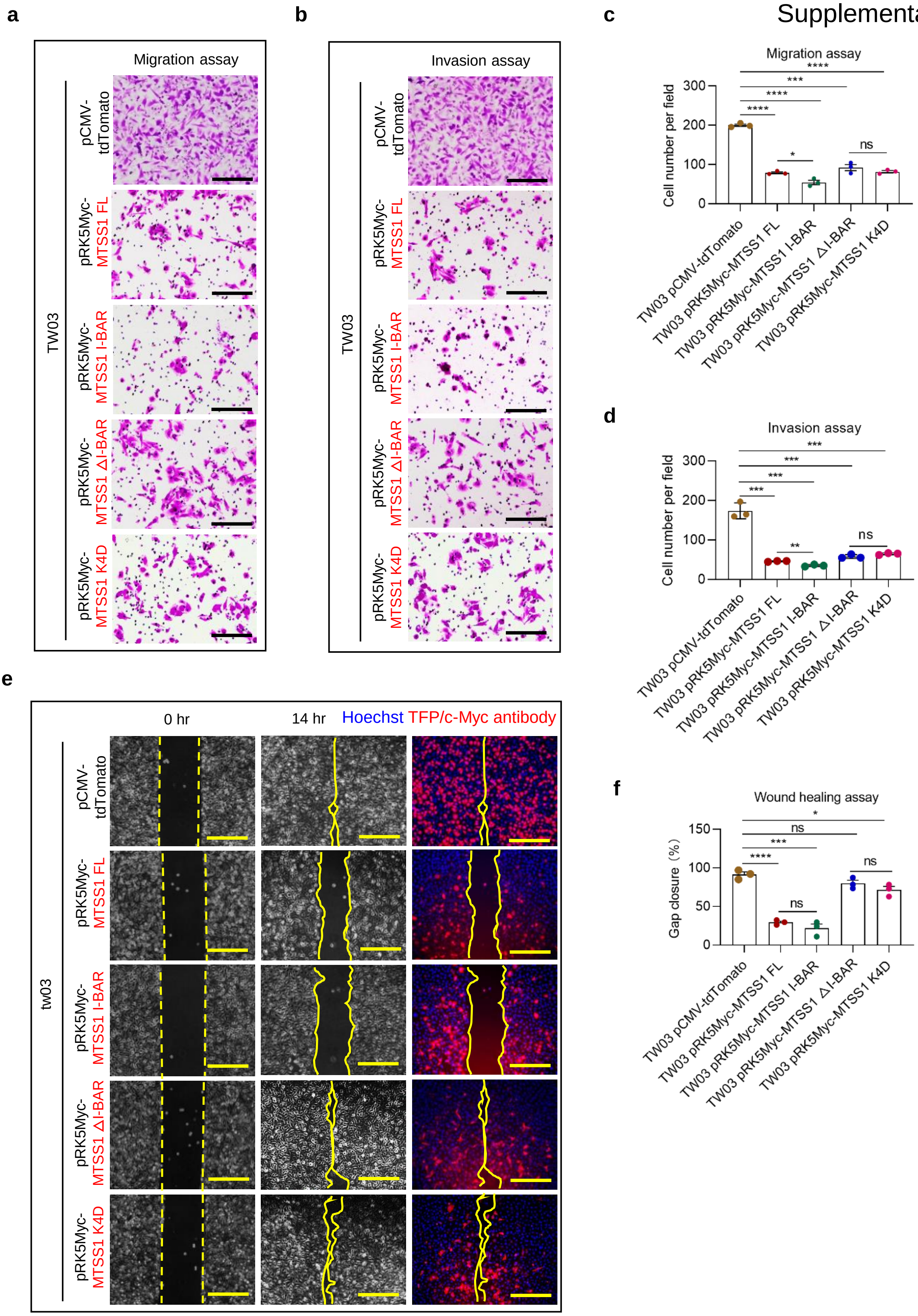

Supplementary Fig.8

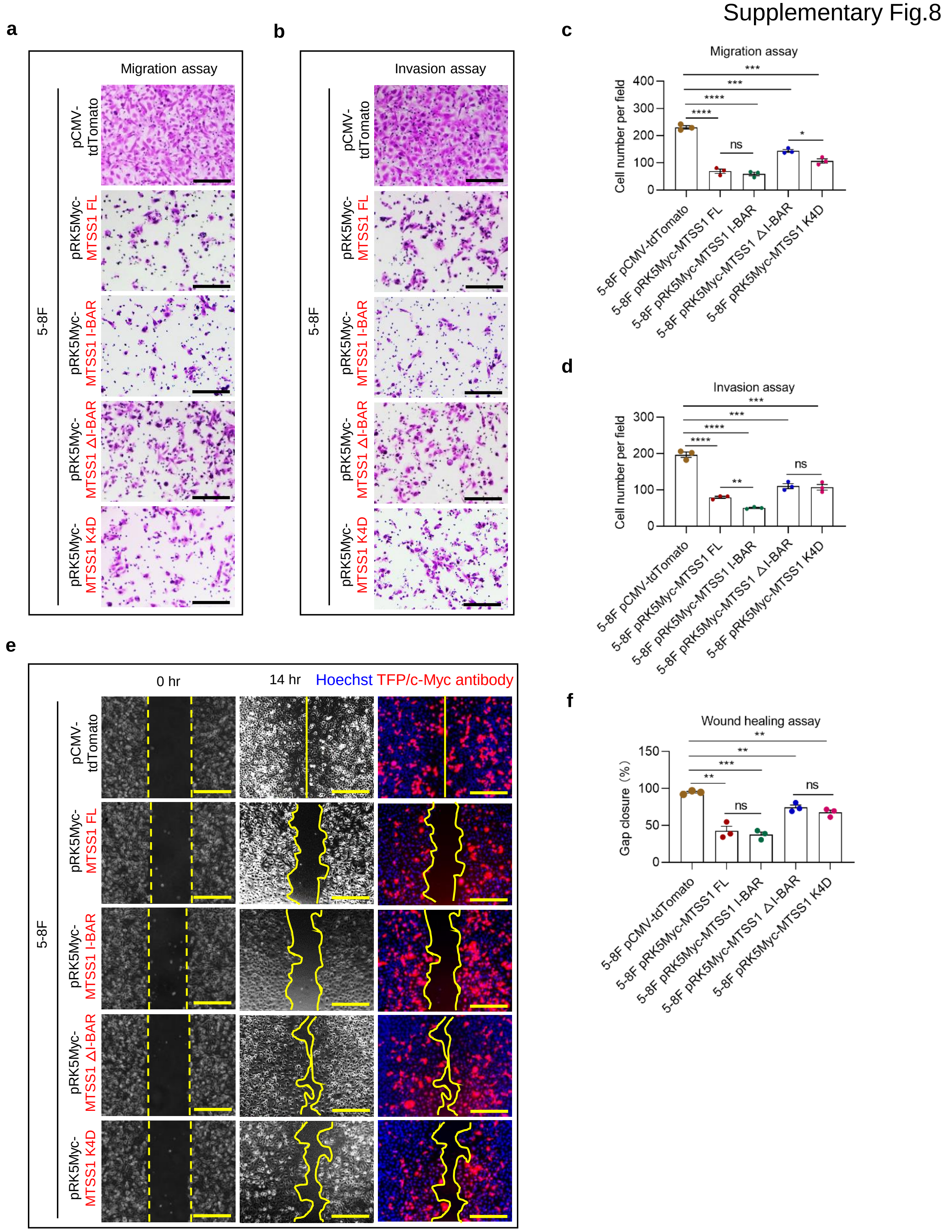

Supplementary Fig.9

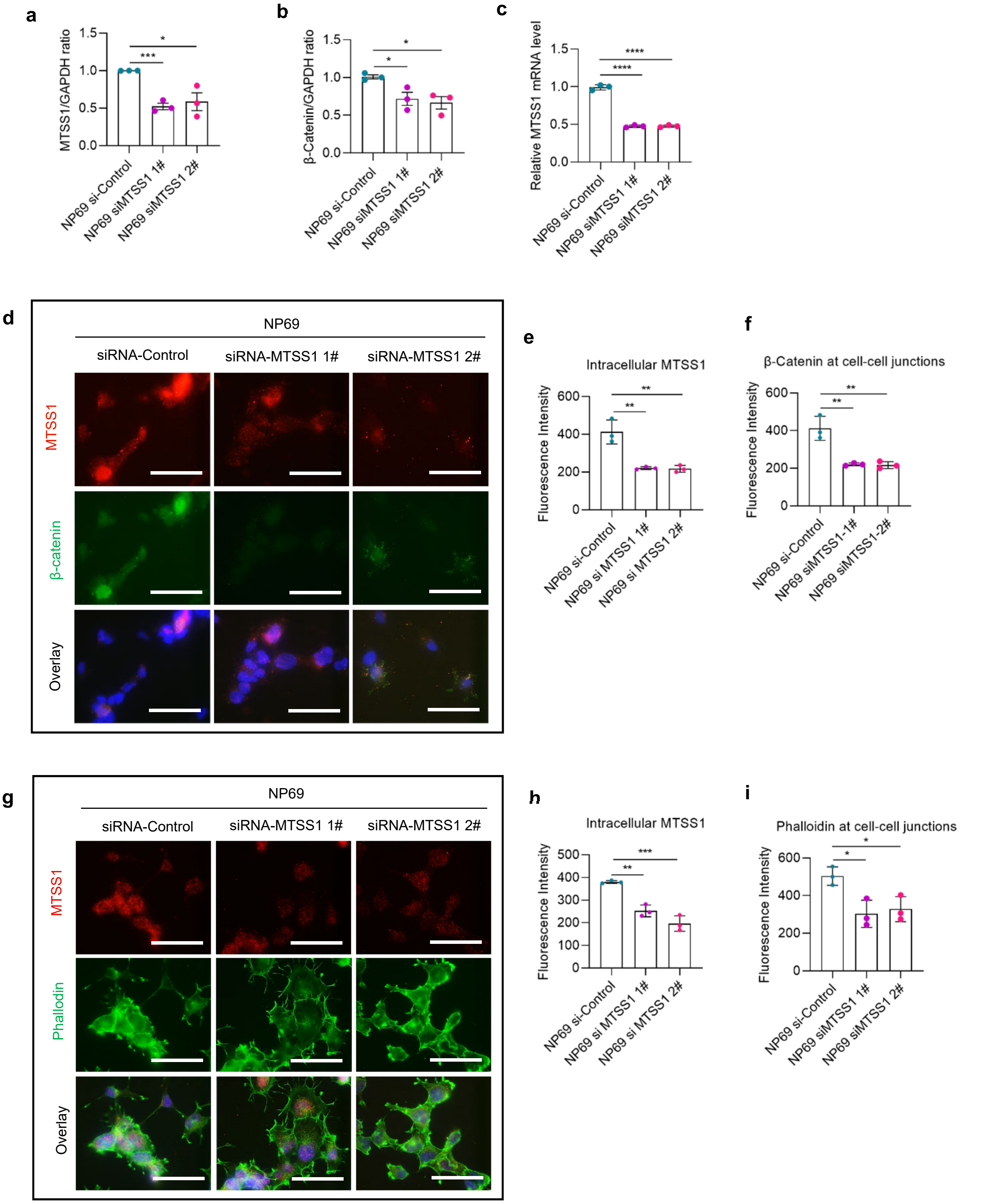

a

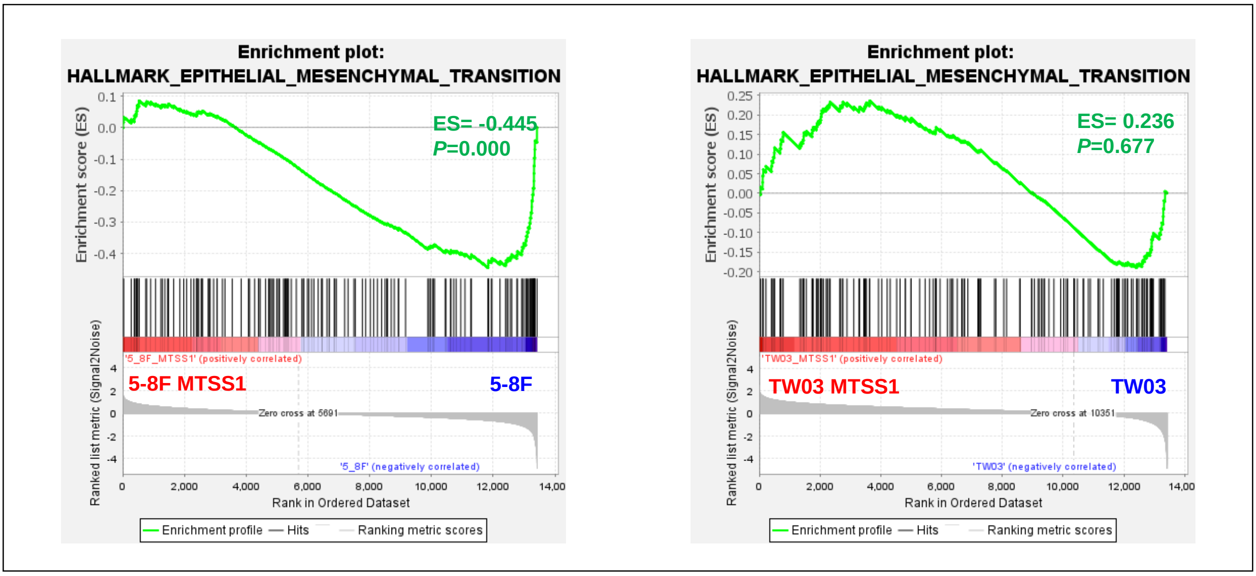
Enrichment plot:
HALLMARK_EPITHELIAL_MESENCHYMAL_TRANSITION
ES= -0.445
P=0.000
5-8F MTSS1
5-8F
Zero cross at 5691
Enrichment score (ES)
Ranked list metric (Signal2Noise)
Rank in Ordered Dataset
Enrichment profile
Hits
Ranking metric scores
Enrichment plot:
HALLMARK_EPITHELIAL_MESENCHYMAL_TRANSITION
ES= 0.236
P=0.677
TW03 MTSS1
TW03
Zero cross at 10351
Enrichment score (ES)
Ranked list metric (Signal2Noise)
Rank in Ordered Dataset
Enrichment profile
Hits
Ranking metric scores

b

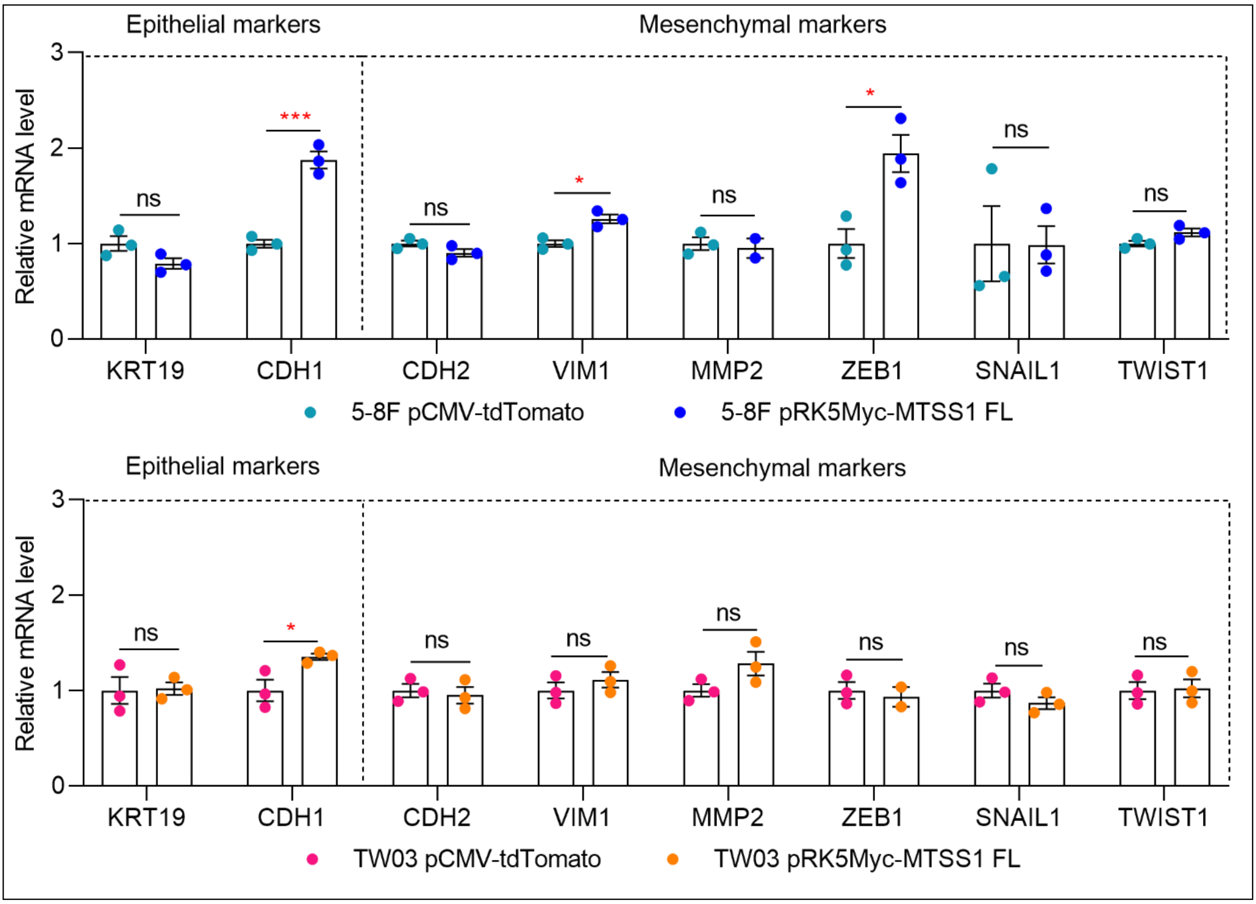
Epithelial markers
Mesenchymal markers
Relative mRNA level
KRT19
CDH1
CDH2
VIM1
MMP2
ZEB1
SNAIL1
TWIST1
5-8F pCMV-tdTomato
5-8F pRK5Myc-MTSS1 FL
Epithelial markers
Mesenchymal markers
Relative mRNA level
KRT19
CDH1
CDH2
VIM1
MMP2
ZEB1
SNAIL1
TWIST1
TW03 pCMV-tdTomato
TW03 pRK5Myc-MTSS1 FL

Supplementary Fig.11
Western Blot analysis of β-catenin phosphorylation levels in response to MTSS1 expression in TW03 cells(Figure3.g)

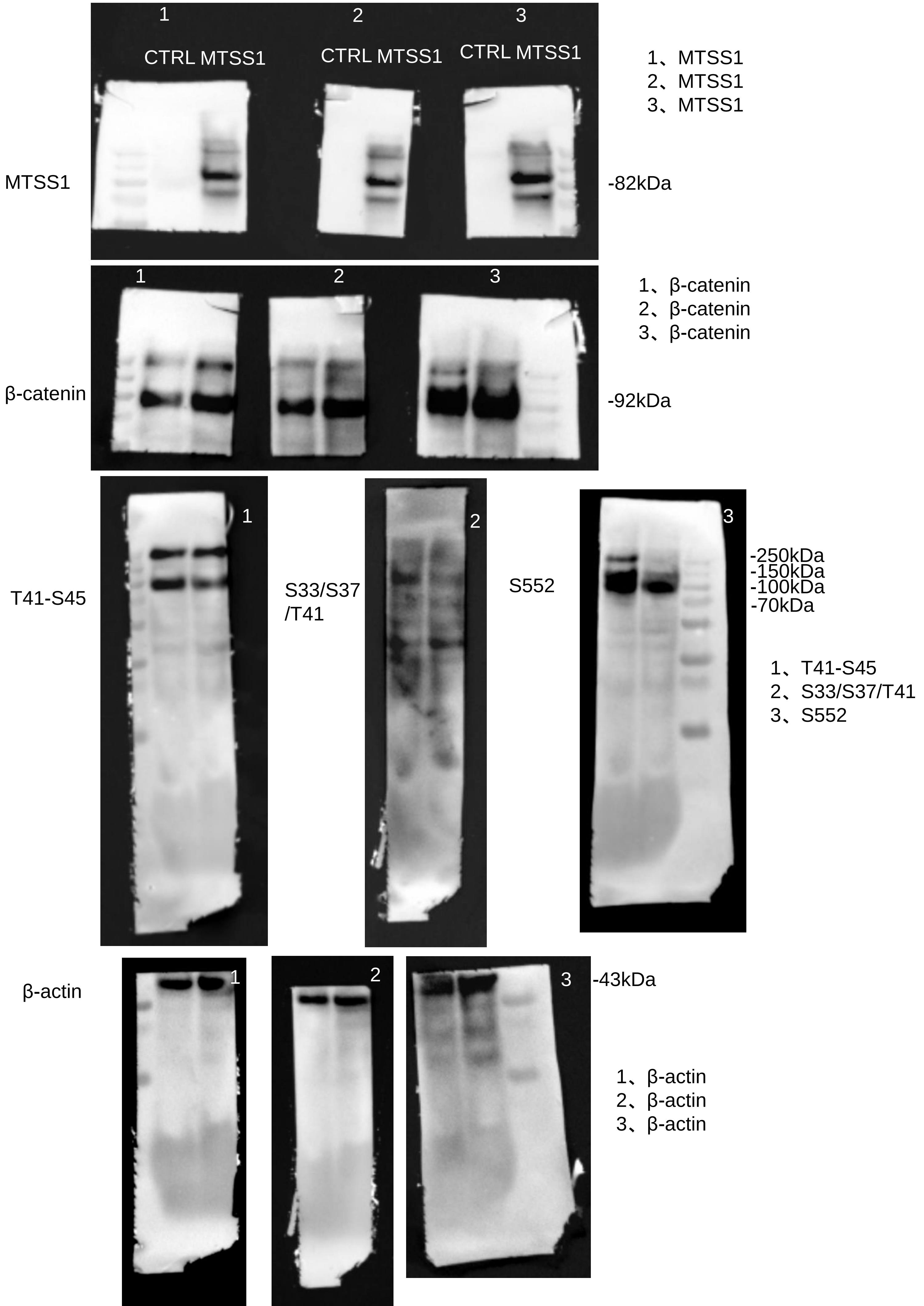

Supplementary Fig.12
Western Blot analysis of MTSS1 expression in two epithelial cell lines (NP69 and 6-10B).(Figure 5a)

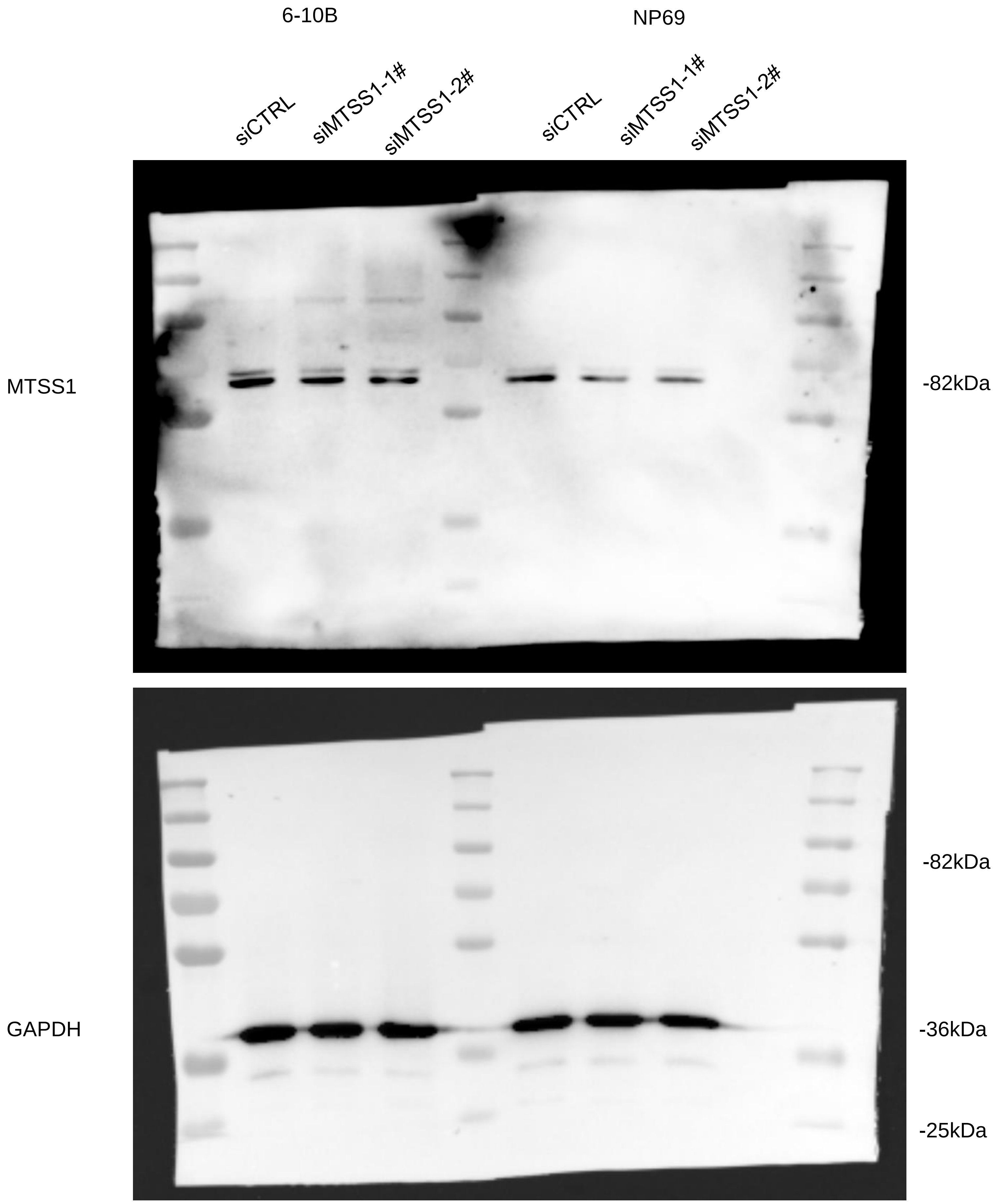

Supplementary Figure 13
Western Blot analysis of MTSS1 expression in two epithelial cell lines (NP69 and 6-10B).(Figure 5a)

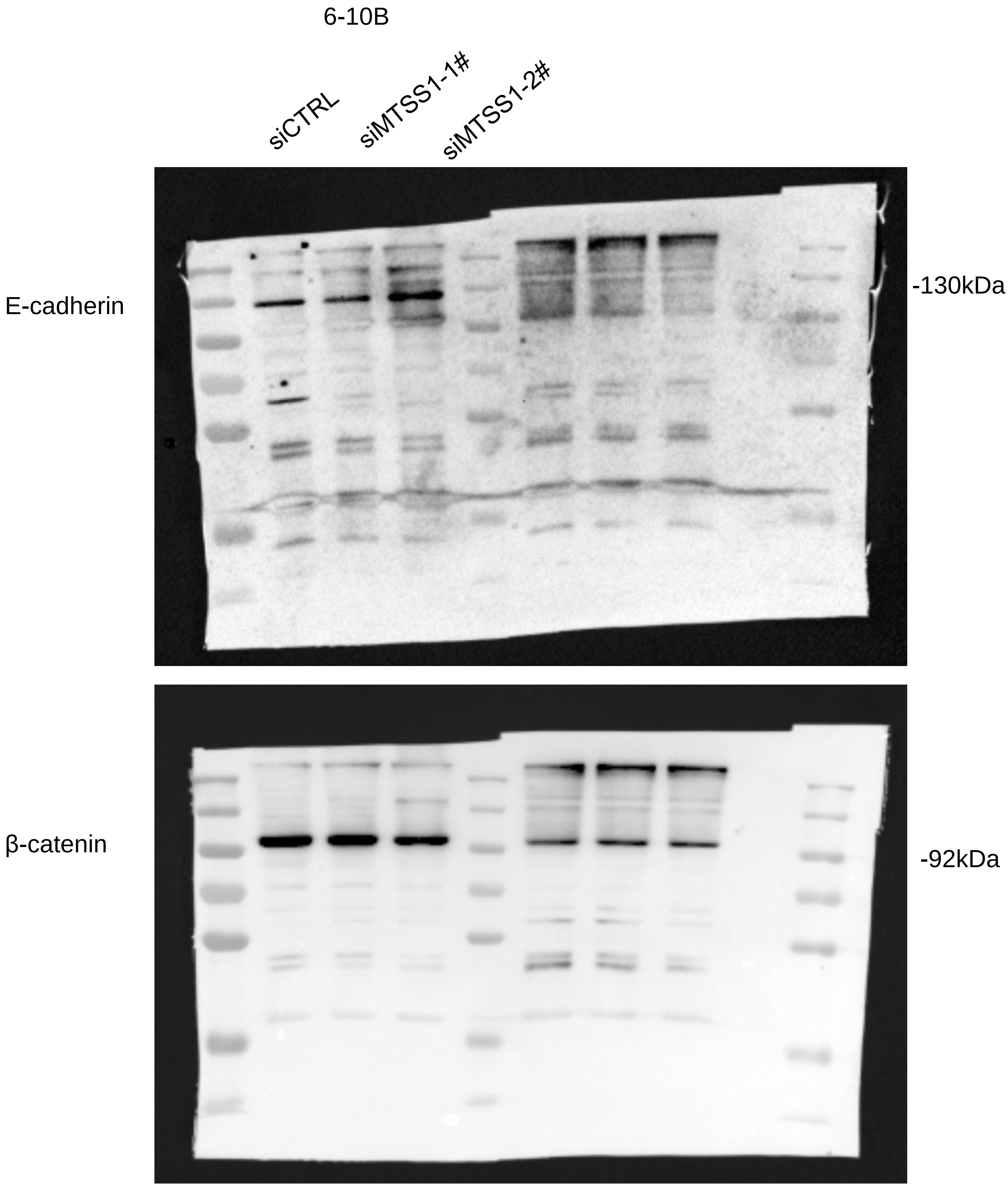

Supplementary Table 1: Key reagents and resources uese in the work.

REAGENT or RESOURCE	SOURCE	IDENTIFIER
**Antibodies**		
Anti-MTSS1 polyclonal antibody	Atlas Antibodies	Cat# HPA075540, RRID:AB_2686756
c-Myc Monoclonal Antibody (9E10)	Thermo Fisher Scientific	Cat# MA1-980-1MG, RRID:AB_2537627
beta Catenin Polyclonal Antibody (CAT-15)	Thermo Fisher Scientific	Cat# 712700, RRID:AB_2533982
E-cadherin Polyclonal Antibody	Thermo Fisher Scientific	Cat# PA5-32178, RRID:AB_2549651
Desmoplakin Recombinant Polyclonal Antibody (14HCLC)	Thermo Fisher Scientific	Cat# 711223, RRID:AB_2633135
JAM-A (CD321) Polyclonal Antibody	Thermo Fisher Scientific	Cat# PA5-19159, RRID:AB_10986745
Claudin 1 Polyclonal Antibody	Thermo Fisher Scientific	Cat# 51-9000, RRID:AB_2533916
Integrin beta4 (H-101) antibody	Santa Cruz Biotechnology	Cat# sc-9090, RRID:AB_2129021
Goat anti-Mouse IgG (H+L) Highly Cross-Adsorbed Secondary Antibody, Alexa Fluor 594	Thermo Fisher Scientific	Cat# A-11032, RRID:AB_2534091
Goat anti-Rabbit IgG (H+L) Highly Cross-Adsorbed Secondary Antibody, Alexa Fluor Plus 488	Thermo Fisher Scientific	Cat# A32731, RRID:AB_2633280
Goat anti-Rabbit IgG (H+L) Cross-Adsorbed Secondary Antibody, Alexa Fluor 647	Thermo Fisher Scientific	Cat# A-21245, RRID:AB_2535813
p-beta-catenin(T41/S45) rabbit polyclonal	Cell Signaling Technology	Cat# 9565
p-beta-catenin(S33/37/T41) rabbit polyclonal	Cell Signaling Technology	Cat# 9561
p-beta-catenin(S552) rabbit polyclonal	Cell Signaling Technology	Cat# 9566
**Biological Samples**		
Healthy adult nasopharyngeal epithelium	Otolaryngology -Head & Neck Surgery, First Affiliated Hospital of Guangxi Medical University (China)	N/A
Human nasopharyngeal carcinoma blocks	Otolaryngology -Head & Neck Surgery, First Affiliated Hospital of Guangxi Medical University (China)	N/A
Human nasopharyngeal carcinoma tissue array	Outdo Bitotech (Shanghai, China)	HNasN132Su01
**Chemicals, Reagents**		

Mycoplasma Detection Kit	Invivogen	Cat#rep-mys-10
Plasmocin® treatment	Invivogen	Cat#ant-mpt
Plasmocin® prophylactic	Invivogen	Cat#ant-mpp
Alexa Fluor™ 488 Phalloidin	Invitrogen	Cat#A12379
Hoechst 33342 Solution (20 mM)	Thermo Fisher Scientific	Cat#62249
Critical Commercial Assays		
RNeasy mini kit (250)	Qiagen	Cat#74106
High capacity cDNA reverse transcription kit with RNase inhibitor	Applied Biosystems	Cat#4374967
FuGENE HD transfection reagent	Promega	Cat#E2311
PowerUp™ SYBR™ Green Master Mix	Applied Biosystems	Cat#A25742
**Data sets**		
GSE12452	GEO	https://www.ncbi.nlm.nih.gov/geo/query/acc.cgi?acc=GSE12452
GSE13597	GEO	https://www.ncbi.nlm.nih.gov/geo/query/acc.cgi?acc=GSE13597
GSE34573	GEO	https://www.ncbi.nlm.nih.gov/geo/query/acc.cgi?acc=GSE34573
GSE40290	GEO	https://www.ncbi.nlm.nih.gov/geo/query/acc.cgi?acc=GSE40290
GSE53819	GEO	https://www.ncbi.nlm.nih.gov/geo/query/acc.cgi?acc=GSE53819
GSE64634	GEO	https://www.ncbi.nlm.nih.gov/geo/query/acc.cgi?acc=GSE64634
GSE68799	GEO	https://www.ncbi.nlm.nih.gov/geo/query/acc.cgi?acc=GSE68799
**Experimental Models: Cell Lines**		
Human: Nasopharyngeal carcinoma 5-8F cells	Our lab	RRID:CVCL_C528
Human: Nasopharyngeal carcinoma 6-10B cells	Our lab	RRID: CVCL_C529
Human: Nasopharyngeal carcinoma TW03 cells	Our lab	RRID:CVCL_6010
Human: Nasopharyngeal carcinoma HK1 cells	Our lab	RRID:CVCL_7084
Human: Nasopharyngeal carcinoma CNE1 cells	Our lab	RRID:CVCL_6888

Human: Nasopharyngeal carcinoma CNE2 cells	Our lab	RRID:CVCL_6889
Human: Nasopharyngeal carcinoma HONE1 cells	Our lab	RRID:CVCL_8706
Human: Nasopharyngeal carcinoma C666-1 cells	Our lab	RRID:CVCL_7949
Immortalised human nasopharyngeal epithelial cell line NP69	Our lab	RRID:CVCL_F755
Immortalised human nasopharyngeal epithelial cell line NP460	Our lab	RRID:CVCL_X205
**qRT-PCR primers**		
GAPDH For 5′-CATCAGCAATGCCTCCTGCAC-3′	Thermo Fisher Scientific	Cat#10336022
GAPDH Rev 5′-GTCATGAGTCCTTCCACGATACCAA-3′	Thermo Fisher Scientific	Cat#10336022
CDH1 For 5′-GCCTCCTGAAAAGAGAGTGGAAG-3′	Thermo Fisher Scientific	Cat#10336022
CDH1 Rev 5′-TGGCAGTGTCTCTCCAAATCCG-3′	Thermo Fisher Scientific	Cat#10336022
MTSS1 For 5'-TCAAGAACAGATGGAAGAATGG-3'	Thermo Fisher Scientific	Cat#10336022
MTSS1 Rev 5'-TGCGGTAGCGGTAATGTG-3'	Thermo Fisher Scientific	Cat#10336022
JAM1 For 5'-GTGAAGTTGTCCTGTGCCTACTC-3'	Thermo Fisher Scientific	Cat#10336022
JAM1 Rew 5'-ACCAGTTGGCAAGAAGGTCACC-3'	Thermo Fisher Scientific	Cat#10336022
CADM1 For 5'-GCTTCTGCTGTTGCTCTTCTCC-3'	Thermo Fisher Scientific	Cat#10336022
CADM1 Rew 5'-GACTTGGCAACTGATGGTCGCA-3'	Thermo Fisher Scientific	Cat#10336022
OCLN For 5'-ATGGCAAAGTGAATGACAAGCGG-3'	Thermo Fisher Scientific	Cat#10336022
OCLN Rew 5'-CTGTAACGAGGCTGCCTGAAGT-3'	Thermo Fisher Scientific	Cat#10336022
CLDN1 For 5'-GTCTTTGACTCCTTGCTGAATCTG-3'	Thermo Fisher Scientific	Cat#10336022
CLDN1 Rew 5'-CACCTCATCGTCTTCCAAGCAC-3'	Thermo Fisher Scientific	Cat#10336022
TJP1 For 5'-GTCCAGAATCTCGGAAAAGTGCC-3'	Thermo Fisher Scientific	Cat#10336022
TJP1 Rew 5'-CTTTCAGCGCACCATACCAACC-3'	Thermo Fisher Scientific	Cat#10336022
DSP For 5'-TGACAGACCGCTGGCAAAGGAT-3'	Thermo Fisher Scientific	Cat#10336022
DSP Rew 5'-CGTGTCAGGTTCTGGAATGGCA-3'	Thermo Fisher Scientific	Cat#10336022
CTNNA1 For 5'-GGACCTGCTTTCGGAGTACATG-3'	Thermo Fisher Scientific	Cat#10336022
CTNNA1 Rew 5'-CTGAAACGTGGTCCATGACAGC-3'	Thermo Fisher Scientific	Cat#10336022
ITGB4 For 5'-AGGATGACGACGAGAAGCAGCT-3'	Thermo Fisher Scientific	Cat#10336022
ITGB4 Rew 5'-ACCGAGAACTCAGGCTGCTCAA-3'	Thermo Fisher Scientific	Cat#10336022
KRT19 For 5′-AGCTAGAGGTGAAGATCCGCGA-3′	Thermo Fisher Scientific	Cat#10336022

KRT19 Rev 5′-GCAGGACAATCCTGGAGTTCTC-3′	Thermo Fisher Scientific	Cat#10336022
ZEB1 For 5′-GGGAGGAGCAGTGAAAGAGA-3′	Thermo Fisher Scientific	Cat#10336022
ZEB1 Rev 5′-TTTCTTGCCCTTCCTTTCTG-3′	Thermo Fisher Scientific	Cat#10336022
SNAIL1 For 5′-TGCCCTCAAGATGCACATCCGA -3′	Thermo Fisher Scientific	Cat#10336022
SNAIL1 Rev 5′-GGGACAGGAGAAGGGCTTCTC -3′	Thermo Fisher Scientific	Cat#10336022
TWIST1 For 5′-GCCAGGTACATCGACTTCCTCT-3′	Thermo Fisher Scientific	Cat#10336022
TWIST1 Rev 5′-TCCATCCTCCAGACCGAGAAGG-3′	Thermo Fisher Scientific	Cat#10336022
VIM1 For 5′-AATCCAAGTTTGCTGACCTCTCTG-3′	Thermo Fisher Scientific	Cat#10336022
VIM1 Rev 5′-TCATTGGTTCCTTTAAGGGCATCC-3′	Thermo Fisher Scientific	Cat#10336022
CDH2 For 5′-ACAGTGGCCACCTACAAAGG-3′	Thermo Fisher Scientific	Cat#10336022
CDH2 Rev 5′-CCGAGATGGGGTTGATAATG-3′	Thermo Fisher Scientific	Cat#10336022
MMP2 For 5′-AGCGAGTGGATGCCGCCTTTAA-3′	Thermo Fisher Scientific	Cat#10336022
MMP2 Rev 5′- CATTCCAGGCATCTGCGATGAG-3′	Thermo Fisher Scientific	Cat#10336022
**Recombinant DNA**		
myc-tagged human MTSS1 FL (pRK5Myc-MTSS1, amino acid 1-759)	kindly provided by Laura M. Machesky (Beatson Institute, Glasgow, UK).	N/A
myc-tagged human MTSS1 K4D (pRK5Myc-MTSS1 K4D, mutant K149,150,152,153D)	kindly provided by Laura M. Machesky (Beatson Institute, Glasgow, UK).	N/A
myc-tagged human MTSS1 △I-BAR (pRK5Myc-MTSS1 , amino acid 235-759)	kindly provided by Laura M. Machesky (Beatson Institute, Glasgow, UK).	N/A
myc-tagged human MTSS1 I-BAR (pRK5Myc-MTSS1 , amino acid 1-254)	kindly provided by Laura M. Machesky (Beatson Institute, Glasgow, UK).	N/A
tdTomato-N1 (pCMV-tdTomato)	Addgene	Cat#54642
pCMV-Myc	Addgene	Cat#631604
**SiRNA**		
siMTSS1(s18915,s18916)	Ambion	Cat#4392420
siRNA negative control No.1	Ambion	Cat#4390843

Software and Algorithms		
ZEN 3.1 blue edition	ZEISS	https://www.zeiss.com/microscopy/int/pr oducts/microscope-software/zen.html
Stream View	OLYMPUS	https://www.olympus- ims.com/en/downloads/detail/?0[downloads][id]=276828114
Revman Manager 5.4	Cochrane	RRID:SCR_003581
GraphPad Prism 9.0	GraphPad Software	RRID:SCR_002798
Photoshop CS	Adobe	RRID:SCR_014199
Image J	NIH	RRID:SCR_003070
Gene Set Enrichment Analysis (GSEA)	Broad Institute	RRID:SCR_003199
The Database for Annotation, Visualization and Integrated Discovery (DAVID)	NCI-Frederick	RRID:SCR_001881

Supplementary Table 2: Collected clinical characteristics of the patients with nasopharyngeal carcinoma

**Clinical characteristic**	**Units**	**MTSS1 expression**			***P* value**
		**High**	**Medium**	**Low**	
Number	n	44	43	44	-
Mean age ±SD	Years	47.16 ±11.78	45.97±10,99	51.47 ±10.64	0.056†
Gender	n (%)				
Male		31 (70.45)	31(72.09)	38 (86.36)	0.156‡
Female		13 (29.55)	12(27.91)	6 (13.64)	
Pathology	n (%)				
Undifferentiated		36 (81.82)	38(88.37)	39 (88.64)	0.861‡
Differentiated		7 (15.91)	4(9.30)	4 (9.09)	
Keratinized		1 (2.27)	1(2.33)	1 (2.27)	
Cervical lymph node metastasis	n (%)				
Yes		28 (63.64)	34(79.079	33 (75.00)	0.246‡
No		16 (36.36)	9(20.93)	11 (25.00)	
Distant metastases	n (%)				
Yes		16 (36.36)	11(25.58)	15 (34.09)	0.291‡
No		25 (56.82)	23(53.49)	20 (45.45)	
Unknown		3 (6.82)	9(20.93)	9 (20.45)	
Clinical stage (Early)	n (%)				
I		5 (11.36)	3(6.98)	7 (15.91)	0.968‡
II		18 (40.91)	18(41.86)	20 (45.45)	
Clinical stage (Advanced)	n (%)				
III		18 (40.91)	13(30.23)	8 (18.18)	0.035‡
IV		3 (6.82)	9(20.93)	9 (20.45)	

† Student's t-tests

‡ Chi-squared tests
